# Phase Difference between Model Cortical Areas Determines Level of Information Transfer

**DOI:** 10.3389/fncom.2017.00006

**Published:** 2017-02-09

**Authors:** Marije ter Wal, Paul H. Tiesinga

**Affiliations:** Department of Neuroinformatics, Donders Institute, Radboud UniversityNijmegen, Netherlands

**Keywords:** oscillations, communication through coherence, synchrony, phase difference, information transfer, PING, multiplexing

## Abstract

Communication between cortical sites is mediated by long-range synaptic connections. However, these connections are relatively static, while everyday cognitive tasks demand a fast and flexible routing of information in the brain. Synchronization of activity between distant cortical sites has been proposed as the mechanism underlying such a dynamic communication structure. Here, we study how oscillatory activity affects the excitability and input-output relation of local cortical circuits and how it alters the transmission of information between cortical circuits. To this end, we develop model circuits showing fast oscillations by the PING mechanism, of which the oscillatory characteristics can be altered. We identify conditions for synchronization between two brain circuits and show that the level of intercircuit coherence and the phase difference is set by the frequency difference between the intrinsic oscillations. We show that the susceptibility of the circuits to inputs, i.e., the degree of change in circuit output following input pulses, is not uniform throughout the oscillation period and that both firing rate, frequency and power are differentially modulated by inputs arriving at different phases. As a result, an appropriate phase difference between the circuits is critical for the susceptibility windows of the circuits in the network to align and for information to be efficiently transferred. We demonstrate that changes in synchrony and phase difference can be used to set up or abolish information transfer in a network of cortical circuits.

## Introduction

Evidence for oscillatory neural activity is found throughout the brain, in several frequency bands and both at the single neuron as well as the network level. Oscillations are linked to a wide range of higher level cognitive functions (Buzsáki and Draguhn, 2004), such as attention (Steinmetz et al., 2000; Gregoriou et al., 2009; Bosman et al., 2012; Saalmann et al., 2012), memory (Fujisawa and Buzsáki, 2011; Watrous et al., 2013), and rule representation (Buschman et al., 2012), as well as circuit level computations such as input selection (Börgers et al., 2008) and tuning (Womelsdorf et al., 2012; Moldakarimov et al., 2014). Over the course of the last two decades, considerable insight has been gained into the generation of oscillatory activity at the level of single neurons and neural circuits (Tiesinga and Sejnowski, 2009; Wang, 2010; Buzsáki and Wang, 2012). However, a mechanistic link between oscillations and high-level processing in the brain remains to be elucidated. The temporal structure of oscillations has inspired a range of hypotheses in which oscillations provide a dynamic coding scheme (Buzsáki and Chrobak, 1995; Fries et al., 2007). Spike timing relative to the background oscillation can carry information [phase coding (McLelland and Paulsen, 2009)] or signal which ensemble of features the coded information belongs to [temporal binding (Singer, 1999)]. Other hypotheses propose that synchronized oscillations act to change the communication between parts of the brain in a dynamic fashion, a view that has received considerable experimental and theoretical support in recent years. The communication hypotheses come in two flavors: (1) oscillations are tags, binding cells, or circuits into assemblies (Olufsen et al., 2003); (2) oscillations are filters, where a receiving circuit only “listens” to activity with a particular sending phase, a specific frequency or a combination of both (Fries, 2005, 2015; Akam and Kullmann, 2014). The latter finds its motivation in the necessity of “effective” or “selective” communication in the brain. The specialized computations a neural circuit performs often require information from several, but highly specific, sources, which change with the task at hand. Routing of information in the brain is therefore assumed to be a fundamental process, which is context dependent and therefore highly dynamic (Akam and Kullmann, 2014). Switching between synchrony and asynchrony, changing frequency of oscillations, as well as shifting their phases, could potentially provide this dynamic communication structure (Fries, 2005, 2015).

The notion of a synchrony-based communication structure is supported by several lines of experimental research. Even though the evidence is of a correlational nature, the findings indicate a close relation between task dynamics and oscillatory dynamics, with cells and circuits involved in similar tasks having similar oscillatory signatures (Canolty et al., 2010). Such optimal frequency and phase relations precede the onset of information transmission between areas (Womelsdorf et al., 2007). These empirical findings are supported by a growing body of modeling studies, which over the last years have provided compelling evidence that selective communication mediated by synchrony is feasible across a range of models (Akam and Kullmann, 2010; Buehlmann and Deco, 2010; Battaglia et al., 2012; Sancristóbal et al., 2014). Models have also identified some limits and requirements for an synchrony-based dynamic communication structure (Akam and Kullmann, 2012; Rolls et al., 2012). However, the single cell and small circuit dynamics underlying the enhanced or reduced communication between areas is still elusive. Specifically, (1) how does oscillatory activity affect the excitability of single circuits and how does this affect the input-output relation of the circuit?; and (2) how can the synchrony and phase difference between local circuit oscillations be modulated and how do these modulations alter the information transmission between circuits in a network?

Here, we address these questions by studying dynamic effective communication in a biophysically inspired model of two *sending* neural circuits projecting to one *receiving* circuit. We show that the receiver can select its source by matching frequency and/or phase of the ongoing background oscillations with one or both sending circuits. We show that the circuits can represent features of their input in their spiking output simultaneously using three different coding schemes, namely oscillation frequency, phase (timing), and population firing rate, each changing differently with frequency and phase (mis)matching. Our results agree with, and extend previous modeling studies, and account for recent experimental findings and they indicate that synchrony not only allows for a dynamic communication structure, but also creates a medium for multiplexing and parallel coding.

## Methods

### Overview of the model

**Figure 1:**
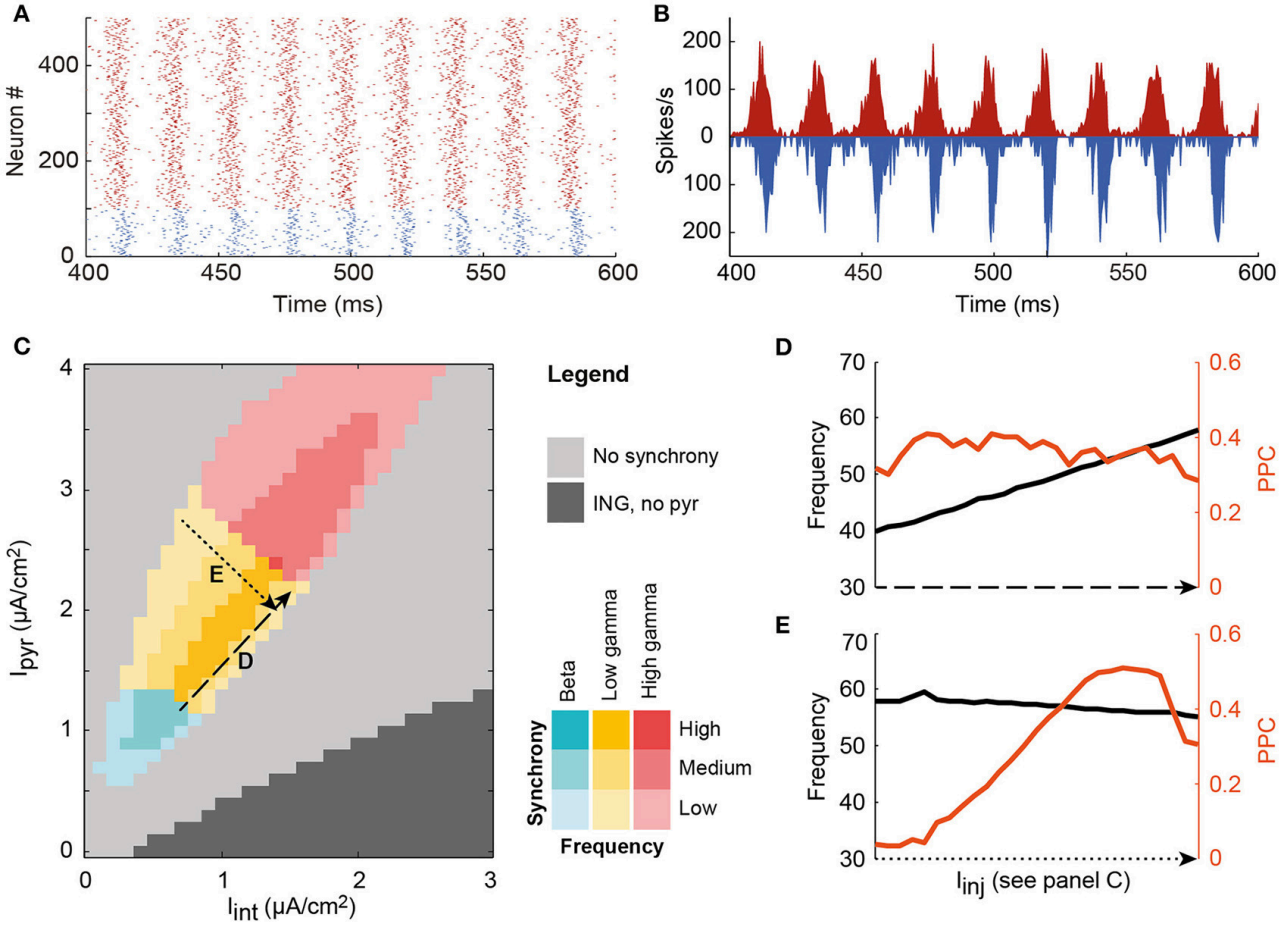
**A single local circuit model shows both asynchronous and PING and ING-generated synchronous network states, depending on the level of input current to the pyramidal cell and interneuron populations**. **(A)** Example rastergram of a synchronous state; **(B)** The corresponding spike density plot of the same simulation run; **(C)** Input to the interneurons (horizontal axis) and the pyramidal cells (vertical axis) determines the level of synchrony of the circuit, indicated by color saturation, and the oscillation frequency, indicated by hue, in a systematic way. In the dark gray area, the circuit synchronized according to the ING mechanism. In the colored area, the PING mechanism synchronized the neurons. In this region of interest, oscillation frequency (black) is increased by increasing the input to both cell types **(D)**, while synchrony (PPC, orange) is changed by decreasing the depolarization of one of the cell types, and increasing the input to the other cell type **(E)**.

**Figure 2:**
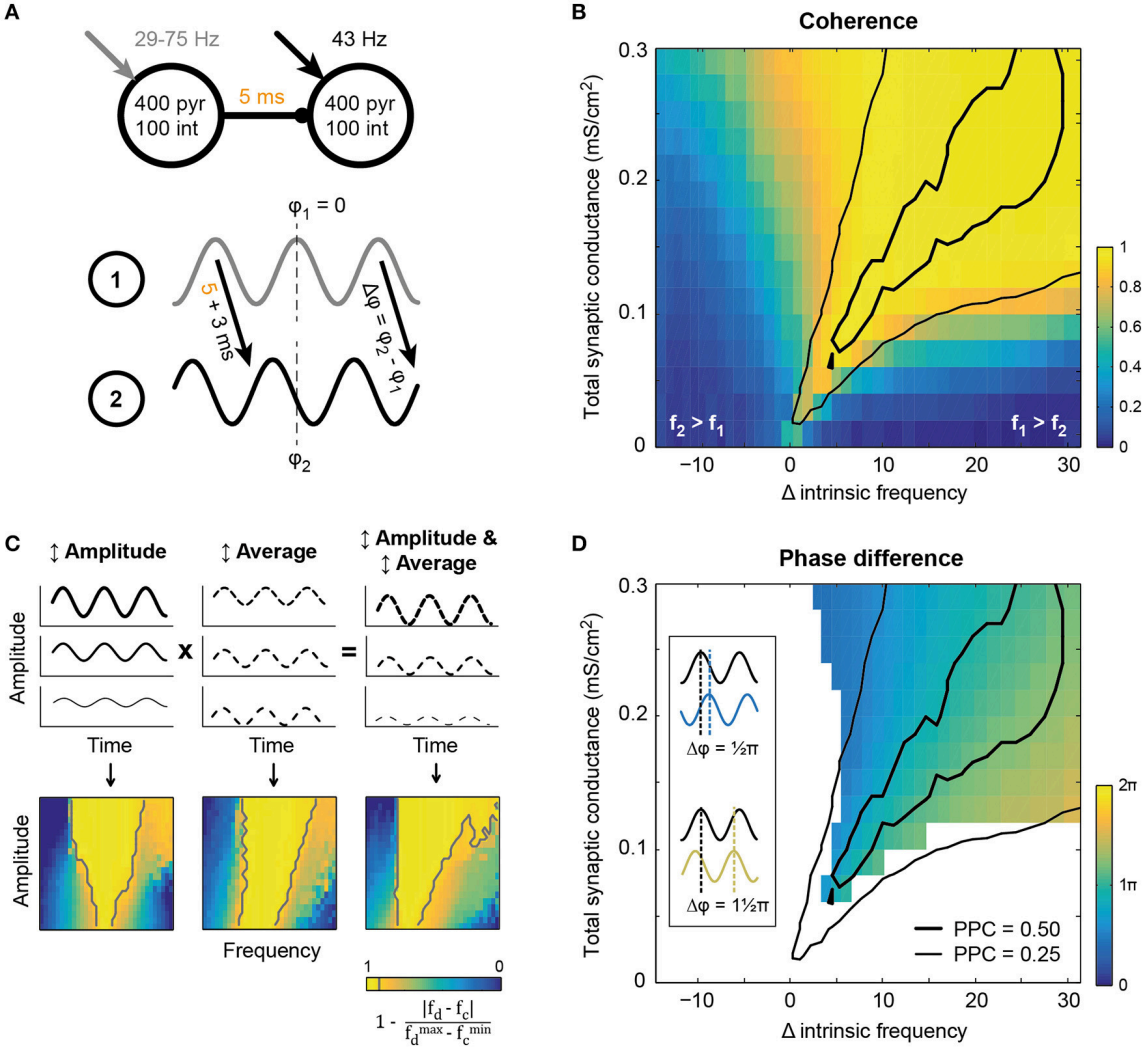
**Synchronization between two circuits in a feedforward network emerged within a tilted Arnold tongue**. **(A)** Schematic of the model setup. Two circuits showing intrinsic oscillatory activity in isolation, were connected by excitatory unidirectional synaptic connections with an axonal delay of 5 ms. The receiving circuit oscillated at 43 Hz, while the oscillation frequency of the sender was varied (x-axis in **B,D**) by increasing the static external drive between runs. **(B)** Coherence (color coded) between the circuits at the oscillation frequency of the sending circuit, showed resemblance to a tilted Arnold tongue. The area of high LFP-LFP coherence coincided with an area of intermediate (thin black line) and high (thick black line) spike-LFP phase consistency between the circuits (that is, spikes from circuit 2 and the LFP from circuit 1, see also Figures SI4C,D). **(C)** Simplified model in which a single circuit was driven by an oscillatory drive of which the frequency was varied together with either the amplitude (left) or the average current (middle). For comparison with panel **B**, “coherence” between drive frequency (*f*_*d*_) and circuit frequency (*f*_*c*_) is plotted, as defined in the equation below the colorbar. Gray lines show the 0.90-contours. Amplitude modulation gave rise to a conventional Arnold tongue synchronization, while offset modulation shifted the intrinsic frequency of the circuit and hence tilted the axis of synchronization. **(D)** Phase difference of the inter-circuit projection, as defined in the bottom panel of **A**, for conditions of high coherence (≥ 0.90). Conventions as in panel **B**, with the same PPC curves for reference.

**Figure 3:**
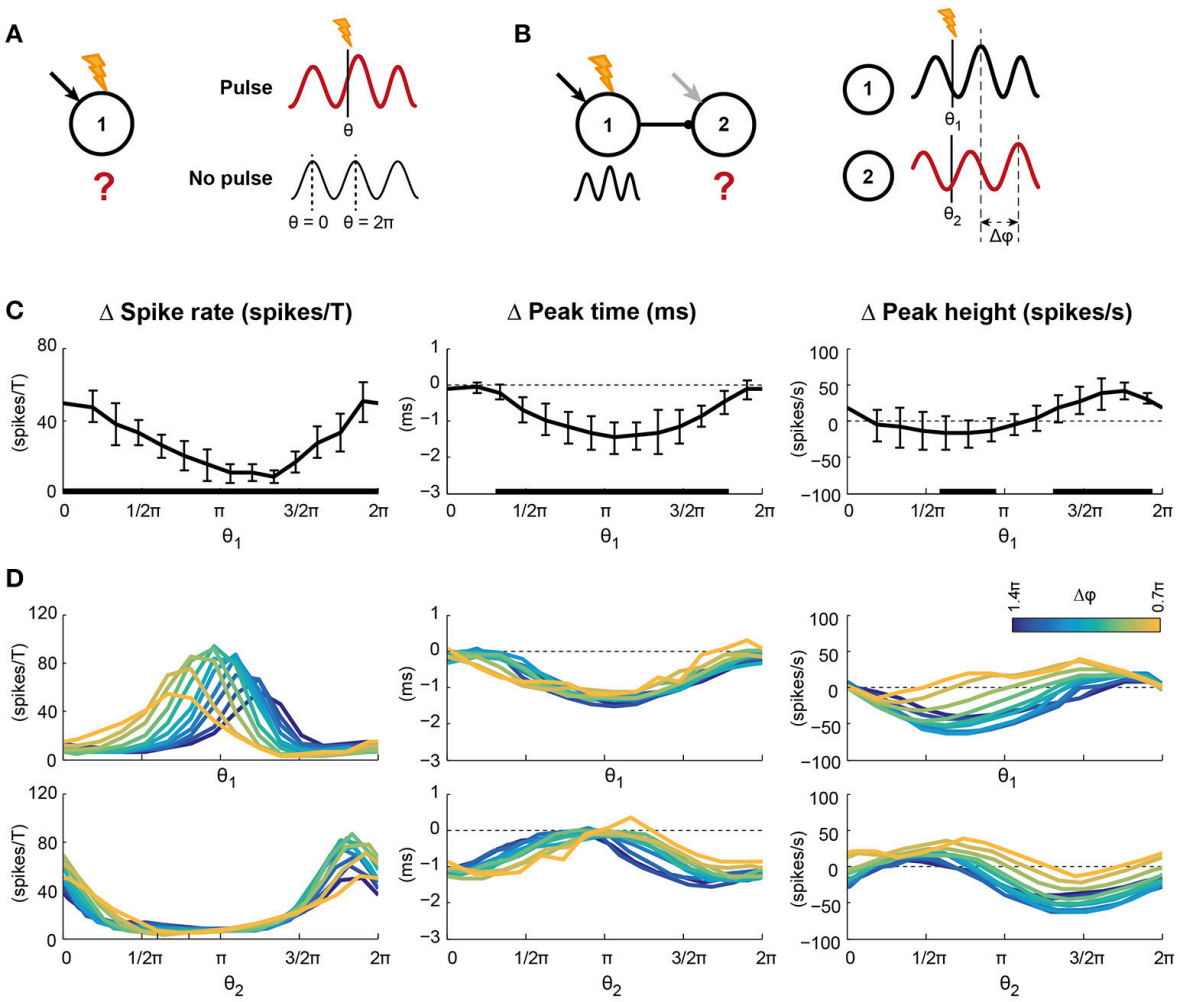
**Pulsed inputs caused changes in spike rate and the features of the oscillation, the magnitude of which depends on the pulse arrival time relative to the ongoing oscillation**. **(A)** Pulses were applied to 75% of the pyramidal cells in circuit 1. The arrival time relative to the ongoing oscillation is indicated by θ, with 0 and 2π indicating the peaks of the oscillation. The effect of a pulse was determined by comparing traces after a pulse with the same condition without pulse presentation. **(B)** Conventions for panel **D**, where pulses were applied to circuit 1 of a feedforward network. **(C)** Changes in spike rate per oscillation period (left), peak time (middle), and height (right) of the first excitatory volley in circuit 1 after pulse presentation. Error bars show standard deviations over 10 simulation runs. Black bars at the abscissa indicate significant deviation from 0. The abscissa indicate pulse arrival phase. **(D)** The spike density traces of circuit 2 were also affected by pulse application to circuit 1, but in addition to the pulse arrival time this effect could depend on phase difference between the two circuits (Δφ, color coded). The two rows show the same data; the top row shows the data relative to the pulse arrival phase in circuit 1, while the bottom row shows the data relative to the pulse arrival phase in circuit 2 (Note: The pulse was applied only to circuit 1). In circuit 2, as in circuit 1, the number of spikes per period and the peak height were modulated by pulse arrival phase, but the size of the modulations depended on the phase difference between the circuits. Peak times of circuit 2 were modulated by pulse arrival phase, but not phase difference. Statistics for the data in D can be found in the main text and SI Figure 7.

**Figure 4:**
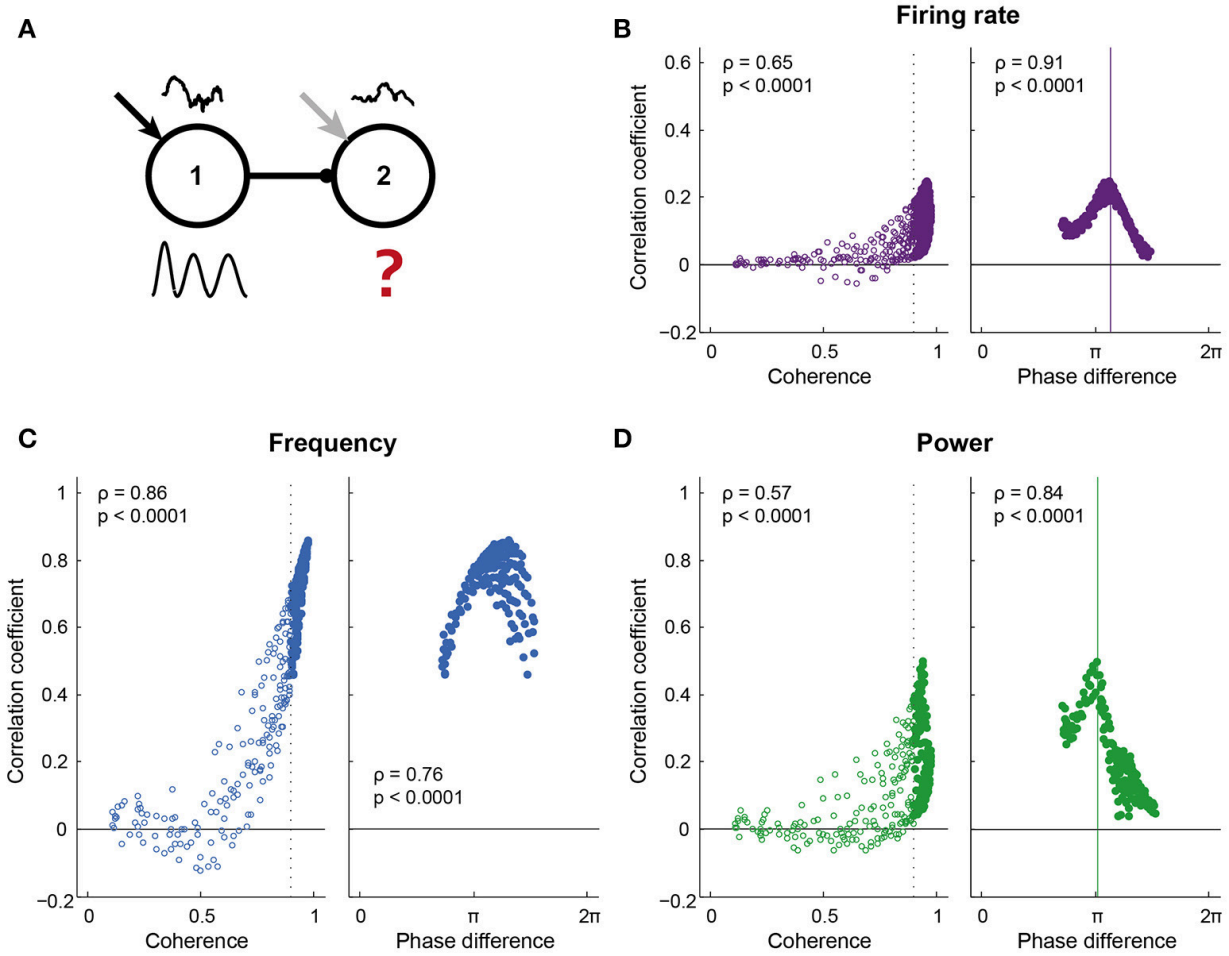
**Information transfer required high coherence and a “good” phase relation**. **(A)** Pyramidal cells in both circuits of the network received a colored white noise current (correlation time = 200 ms), which was identical within each circuit, but uncorrelated across circuits. Correlating the instantaneous spike density frequency, power and spike rate of the two circuits gave a measure of information transfer: 1 indicates that all variability in the receiving circuit is explained by the sender (a high level of information transfer), while 0 indicates a low level of information transfer. **(B–D)** show the correlation coefficient for a range of coherence and phase conditions, for spike density frequency **(B)**, power **(C)**, and firing rate **(D)**. For frequency, the correlation coefficient increased predominantly with coherence between the circuits. For power and firing rate, the range of correlation values was broad for conditions of high coherence, with strong correlations linked to a “good” phase difference between the circuits. Phase impacted the correlation coefficient for frequency, but to a much smaller extent than for power and firing rate.

**Figure 5:**
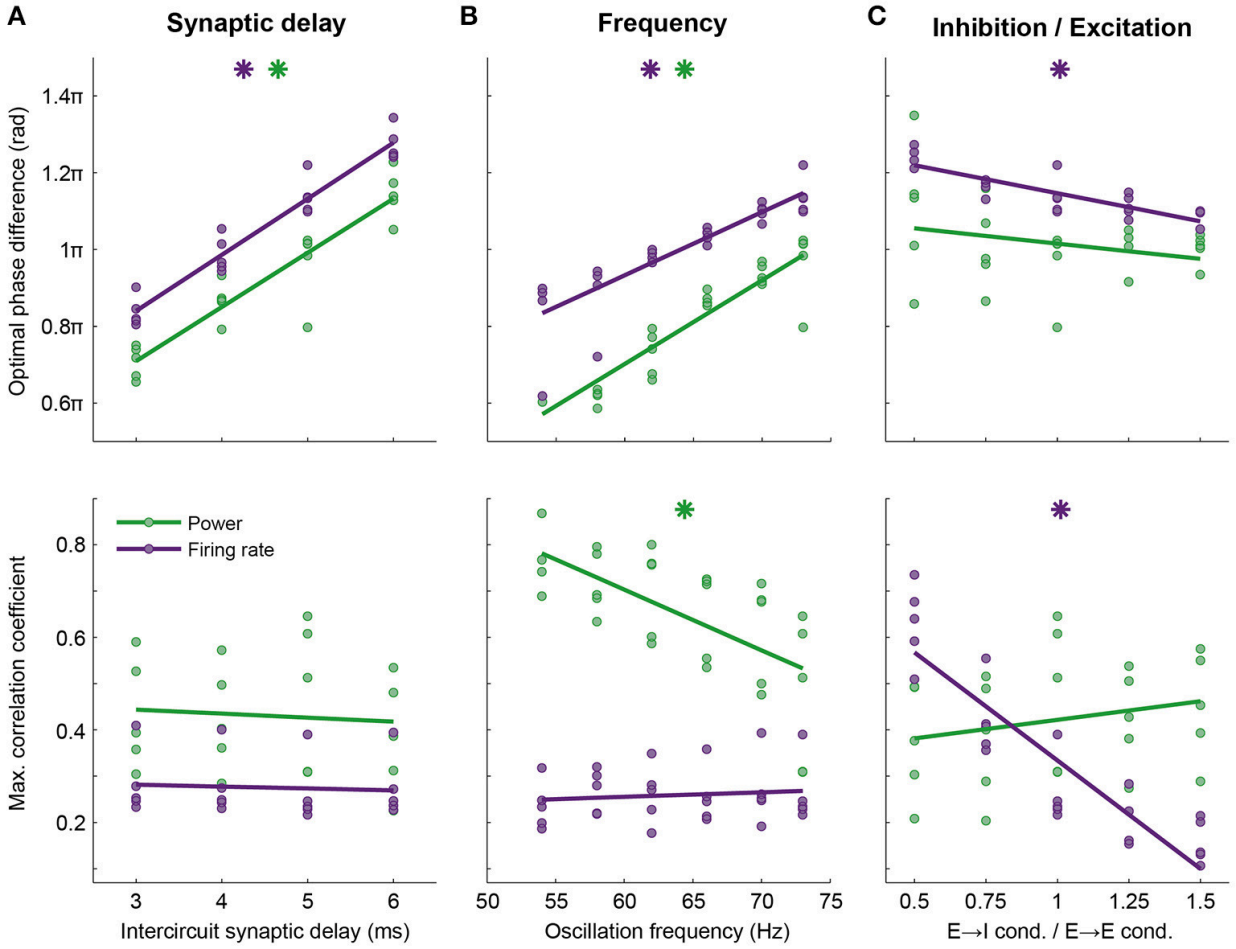
**Optimal phase difference (top row) and information transfer at this phase (bottom row) depend on network properties such as axonal delay, frequency and balance between excitation and inhibition**. Here, data are shown for firing rate (purple) and power at the oscillation frequency (green, compare to Figure 4B). Every point shows data from one simulation, lines are linear fits. The phase of optimal information transfer depended on the characteristic of the network: The synaptic delay between the circuits **(A)** and the oscillation frequency **(B)** and to a much lesser extent, the E → I conductance relative to the E → E conductance **(C)**. Other parameters (synaptic conductance, connection probability, etc.) were kept constant. Stars indicate significant correlations. The level of information transfer at the optimal phase strongly depended on the E → I—E → E conductance ratio for firing rate, but not power; **(C)** stronger projection to E cells in the receiving circuit led to more effective communication of firing rates. Information transfer through power was frequency dependent: Lower frequencies performed better **(B)**. Parameters used in Figure 4B: 5 ms delay, an oscillation frequency of 73 Hz and an E → I—E → E conductance ratio of 1.

**Figure 6:**
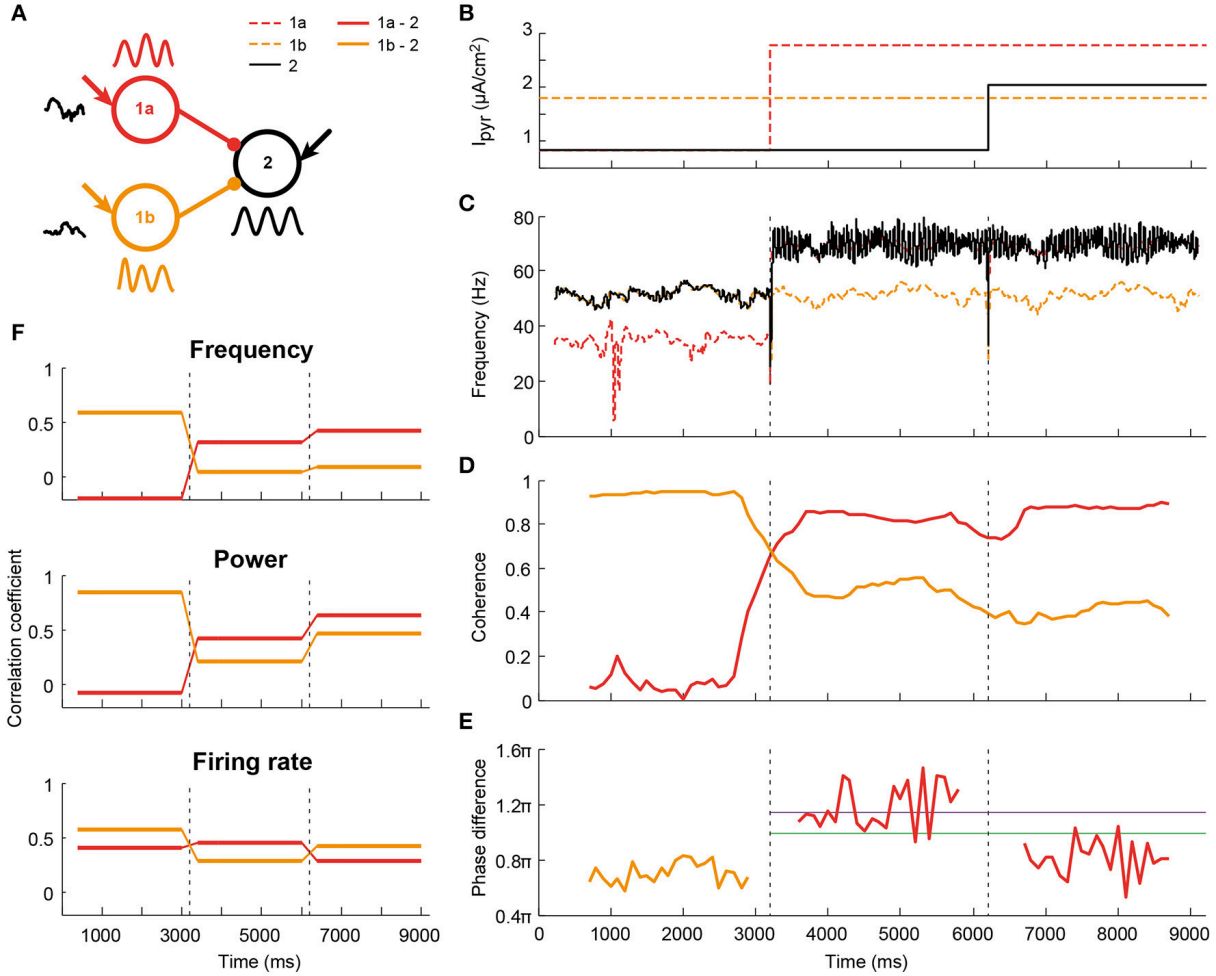
**Illustration (one simulation) of altered communication in a network comprised of two senders and one receiver as a result of coherence and phase changes**. **(A)** Schematic of the network: two circuits project to a third one with identical connection parameters. Both senders received independent noise currents. In addition, all circuits receive a static depolarization, which could change after 3000 ms-long epochs, as indicated in **(B)**; **(C)** The changes in depolarization levels altered the oscillation frequency of the circuits. As a result, the receiving circuit switched from being synchronized to the sender 1b (orange), to being synchronized to sender 1a (red); **(D)** The coherence levels changed accordingly; **(E)** For the coherent pairs, phase differences were calculated. Both coherence and phase were time-resolved by taking 1000 ms windows, spaced 100 ms apart. The purple and green lines corresponded to the peak phases of firing rate and power, respectively, see Figure 4. **(F)** Information transfer was assessed, as before, by taking the frequency, power and firing-rate-per-period traces per epoch of 3000 ms, and correlating these between circuits. The information transfer from sender 1b to the receiver was reduced as this connection lost its coherence in the second epoch. Concomitantly, communication between 1a and the receiver increased after this coherence switch. In epoch 3 the firing rate transfer was reduced due to a less favorable phase relationship.

We implemented a model network, consisting of local circuits representing one (Figure 1), two (Figures 2–**5**), or three (**Figure 6**) different brain areas. Each local circuit was made up of 500 spiking neurons. We used well-established conductance-based single compartment models for fast-spiking interneurons (Wang and Buzsáki, 1996) and pyramidal cells (Golomb and Amitai, 1997). Equations for both neuron types and detailed parameter settings can be found in the Supplemental Information. A local circuit contained 400 pyramidal cells and 100 interneurons, consistent with the ratio found in cortex (Markram et al., 2004). Excitatory and inhibitory synaptic connections within a circuit were modeled using AMPA and GABA kinetics, respectively (see Supplemental Information). Connections between circuits were purely excitatory, but projected to both cell types. Connections were made randomly, according to a connection probability and with a synaptic strength, which depended on both the pre- and post-synaptic cell types and reflected anatomical data (Table 1). Connections within a circuit had synaptic delays of 1 ms and connections between circuits had 5 ms delays.

**Table 1:** **Connection probabilities and unitary synaptic strengths for the connections in the network model**.

**Connection type**	**Connection probability (%)**	**Unitary synaptic strength (μS/cm^2^)**
E to E within circuit	10	1.2
E to I within circuit	30	1.0
I tot I within circuit	20	12
I to E within circuit	60	5.0
E to E between circuits	5	0–15
E to I between circuits	10	0–7.5

*The probabilities and synaptic strengths were inspired by experimental findings (Holmgren et al., 2003; Thomson and Bannister, 2003; Binzegger et al., 2004; Markram et al., 2004; Fino and Yuste, 2011)*.

The model was implemented in Matlab (MATLAB, 2014). The differential equations were integrated using a fourth order Runge-Kutta algorithm, with a time step of 0.05 ms. All simulations were repeated with different random number generator seeds to assure that the results presented were representative for the system studied. With the exception of the examples (**Figures 1A–E** and **6** of the main text and SI Figures 1A, SI8, 11), all other figures report the average over these runs. Each data point in **Figure 5** and the SI figures are averages of 5 runs, while the remainder of the figures report averages of 10 runs. Differences between runs started from different seeds of the random number generator were very small and are reported, when present, in the text and in the Supplemental Information.

### Input to the circuits

All neurons were activated by injecting a constant depolarizing current: *I*_inj_ = *I*_0_ + *I*_σ_, where *I*_0_ is a tonic current that is common to all neurons of a given type in a circuit and *I*_σ_ is a tonic current that differed between neurons. For both neuron types *I*_σ_ was drawn from a Gaussian distribution with mean 0 μA/cm^2^ and standard deviation 0.1 μA/cm^2^.

During the simulations conducted for Figure 3 the pyramidal cells received pulses. These pulses mimicked the depolarization caused by a single presynaptic action potential via an AMPA synapse, but could also represent the light-induced opening of channelrhodopsin-channels. The equations for the pulsed inputs are given in the Supplemental Information. In the simulations for Figure 3, 75% of the neurons in circuit 1 received the pulsed input. These neurons were randomly selected. The pulsed input had a phase-dependent effect on the receiving population, as reported in Figure 3. While the amplitude of the effects reported in Figure 3 changed with the number of neurons receiving the pulse (Figure SI6A), the phase-dependency did not.

In the simulations for Figures 4–**6**, a noise current was added to *I*_inj_ of the pyramidal cells. The noise current *I*_noise_ was the same for all pyramidal neurons in a given circuit, but was statistically independent for each circuit. It was constructed by filtering white noise resulting in a frequency spectrum with an approximate 1/f amplitude fall off. The seeds for noise current generation were chosen to produce uncorrelated currents between circuits (for all selected seeds the correlation between currents was below 0.05).

In Figures 3–5, the phase difference between two circuits was manipulated. The phase difference depended on the difference in intrinsic oscillation frequencies of the two circuits, as is described in the section “Synchronization between areas connected with unidirectional projections occurs along a tilted Arnold tongue” and Figure 2. In Figure 2, differences in intrinsic frequency were introduced by applying different levels of depolarization to the neurons in circuit 1. However, this situation is symmetric; phase differences can also be introduced by changing the intrinsic frequency of circuit 2 while keeping the input to circuit 1 fixed, as long as the intrinsic frequency of circuit 2 is lower than that of circuit 1 (section “Synchronization between areas connected with unidirectional projections occurs along a tilted Arnold tongue”). The latter approach was used in Figures 3–6, because it allowed us to use identical inputs to circuit 1 across conditions, and therefore, identical projections from circuit 1 to 2. For both figures, circuit 1 was oscillating at 73 Hz, while the intrinsic oscillation frequency of circuit 2 was varied between 29 and 75 Hz.

### Analysis

#### Spikes and spike density

Spikes were detected by determining when the membrane potential crossed a threshold, set at −20 mV for pyramidal cells and 0 mV for interneurons. Spike times were saved and the spike density *D*_spike_ was calculated for each population using Δ*t* = 0.5 ms bins:

$$\begin{array}{l} D_{\text{spike}}\text{​}\left(t\right)=\frac{1000}{\Delta t\;N}\sum_{i}X_{i}(t)\\ X_{i}\text{​}\left(t\right)=\{\begin{array}{cc} 1 & \text{if }t_{j}^{i}\in [t,t+\Delta t)\\ 0 & \text{otherwise} \end{array} \end{array}$$

Here $t_{j}^{i}$ denotes the time of the j^th^ spike of the i^th^ neuron and N is the total number of neurons in the population. We considered the activity of the interneuron and pyramidal cell populations separately in each circuit.

#### Frequency

The instantaneous oscillation frequency of each circuit was determined from the pyramidal cell spike density trace. Using the interneuron spike density yielded identical results (compare Figures SI2A,B). The frequency was determined using a wavelet transform, performed with the Wavelet Toolbox for Matlab. As a mother wavelet the complex Morlet wavelet with frequency bandwidth *f*_*b*_ of 1 Hz was used:

$$\Psi \text{​}\left(t\right)=\;\frac{1}{\sqrt{\pi f_{b}}}e^{i2\pi t}e^{-t^{2}{╱}_{f_{b}}\;}$$

Frequency analysis was skipped for input conditions where synchrony within a circuit was extremely low (PPC < 0.05, see next paragraph).

#### Within-circuit synchrony

The synchrony of neuronal activity in one circuit was quantified by the pairwise phase consistency (PPC, Vinck et al., 2011, 2012). First, for each spike, the corresponding phase in the pyramidal cell spike density function was determined (see below). Second, for each pair of spikes, within and across neurons, the inner product of the phases in the complex plane was computed. The inner products were averaged, yielding a value between 0 (no consistency, i.e., no synchrony between spikes in one circuit) and 1 (perfect consistency; all spikes in the circuit occur at the same phase). The measure was shown to be unbiased and independent of spike count and can therefore be used to compare within-circuit synchrony across frequencies. For simplification purposes, we refer to PPC values below 0.25 as “low synchrony,” to PPCs between 0.25 and 0.5 as “intermediate synchrony” and to PPC values of 0.5 and over as “high synchrony.” The reported PPCs are based on the pyramidal cell spike density and phase trace. Repeating the analysis for interneurons yielded higher, but qualitatively similar, PPC values (compare Figures SI2C,D), resembling the behavior observed experimentally in cortical circuits (Hasenstaub et al., 2005).

#### Phase and phase difference

The instantaneous phase of a circuit's oscillatory activity was determined by performing a wavelet transform of the pyramidal cell spike density function, as described in the section “Frequency.” The resulting phase traces were normalized to values between 0 and 2π, with 0 assigned to the peak of the oscillation and 2π to the peak of the next period. To obtain the phase difference between two circuits, the unwrapped phase trace of the first circuit was subtracted from the unwrapped trace of the second circuit. For conditions of high synchrony between circuits (coherence ≥ 0.90) the mean value of the instantaneous phase difference gave the phase difference between the circuits. For conditions of low synchrony (coherence < 0.90), the variance of the instantaneous phase difference was high, rendering the mean phase difference meaningless; the mean phase difference is therefore not reported for conditions of low inter-circuit synchrony.

#### Coherence

Coherence between the circuits was obtained from the spike densities of the pyramidal cell populations using the multi-taper coherence method from the open source Matlab toolbox Chronux (Bokil et al., 2010). Multi-taper coherence between two signals *x* and *y* is defined as follows:

$$C_{XY}\text{​}\left(f\right)=\frac{{\left|\sum_{k}X_{k}\text{​}\left(f\right)\;\cdot \text{ ​}Y_{k}^{*}\text{​}\left(f\right)\right|}^{2}}{\sum_{k}{\left|X_{k}\text{​}\left(f\right)\right|}^{2}\sum_{k}{\left|Y_{k}\text{​}\left(f\right)\right|}^{2}}$$

Here, *X*_*k*_(*f*) is the Fourier spectrum of the *k* ^th^ taper of signal *x*. For the multi-taper analysis, the time-bandwidth product was set to 30 and we used 1 s (Figures 2, 6) or 3 s (Figures 4, 5) of simulated data with a sampling rate of 2000 Hz. High coherence was defined as ≥ 0.90, which empirically corresponded well with the conditions in which the frequencies of the circuits were found to be less than 0.05 Hz apart (compare Figure 2B and Figure SI4A).

#### Measures of information

“Information” was added to the input to the neurons by either adding pulsed input (Figure 3, section “Oscillations create windows of high input susceptibility”) or an identical correlated noise current to all pyramidal cells in a circuit (see section “Input to the circuits,” Figures 4–6 and section “Synchrony and good phase relations lead to high information transfer and Phase relations allow for input selection”). We analyzed the effect of these inputs on both spiking and oscillatory activity, and either in short period just after the pulsed inputs, or the entire time trace for noise currents. Short-term spiking effects were assessed by the number of spikes in one oscillation period after pulse onset (to eliminate the modulation of spike rate due to the oscillation). Longer-term effects were assessed by averaging the spike density in windows of one oscillation period, after which the data were smoothed with a $\frac{t\;-\;t_{j}}{\Delta t}$ smoothing kernel with a width of one oscillation period. Effects of pulsed information on frequency were assessed by comparing the timing of the next spike density peak after pulse onset, while ongoing effects were studied using the instantaneous frequency (or equivalently, phase) of the spike density trace. Similarly, short term effects on synchrony were studied using the height of the first peak of the spike density trace after the pulse, and ongoing effects were assessed by the power of the spike density at this frequency.

#### Information transfer

Information transfer was determined by calculating the correlation coefficient between the 3 s long signal traces of the two circuits, for each of the three signal types, firing rate, oscillation frequency and power, as described in the previous section. The correlation coefficient of two signals *x* and *y* is defined as follows:

$$\rho =\frac{\sum_{i}(x_{i}-\bar{x})(y_{i}-\bar{y})}{\sqrt{\sum_{i}{\left(x_{i}-\bar{x}\right)}^{2}}\sqrt{\sum_{i}{\left(y_{i}-\bar{y}\right)}^{2}}}$$

Heuristically, a correlation coefficient of 0 indicates that none of the variability in circuit 2 is explained by variability in circuit 1 and hence that no information from circuit 1 was transferred to circuit 2, while a correlation coefficient of 1 indicates a full transmission of noise-induced variability from circuit 1 to circuit 2. We compared this method to a more conventional Mutual Information (MI, 6 bins) approach, and found similar results (compare Figure 4 and Figure SI10). The Correlation and MI approaches are expected to be equivalent, because the signals are dictated by the independent (uncorrelated) colored noise signals, and the signals themselves are normally distributed. A similar approach was used for the instantaneous information transfer shown in Figure 6. In this example, the receiver circuit (2) received inputs from two sending circuits (1a and 1b), which in turn each received a noise current. Coherence and phase between circuits 1a/1b and 2 were changed in two steps, by changing the static depolarizing current to circuits 1a and 3 (see Figure 6B for values). The total synaptic conductance between the senders and the receiver was identical and set to 0.22 mS/cm^2^. The instantaneous coherence, phase and information transfer between 1a and 2 and between 1b and 2 were determined as described above in overlapping 1000 ms windows, spaced 100 ms apart. 400 ms windows around the transitions were excluded from the information transfer analysis.

## Results

### Synchrony and oscillation frequency can be manipulated by cell-type specific depolarization

To study how oscillations could facilitate communication in cortical networks, we developed a model of a local cortical circuit, as described in the Methods section, of which the oscillatory characteristics can be controlled. This single circuit model showed both synchronous and asynchronous behavior (Figure 1). When the neurons in a single circuit received a depolarizing current, the neurons spiked either at random times with respect to each other (light gray area) or synchronized their spike timing to produce oscillatory network activity (dark gray and colored areas). In the dark gray area, pyramidal cells spike rates were low or zero, while the interneurons synchronized due to the interneuron-interneuron interactions, known as the Interneuron Network Gamma (ING) mechanism. In the colored area, connections between pyramidal cells and interneurons were required for synchronization (see Figure SI3), indicating a Pyramidal Interneuron Network Gamma (PING) synchronization mechanism (Whittington et al., 2000; Tiesinga and Sejnowski, 2009). In the PING mechanism, (small) groups of synchronous pyramidal spikes trigger interneuron activity, which in turn shuts down pyramidal cell spiking until inhibition wears off and the cycle starts anew. As a result, PING synchrony has a characteristic firing pattern, in which pyramidal cell volleys precede interneuron volleys by a couple of milliseconds, as is observed in the rastergram and the spike density graph of the local circuit model (Figures 1A,B). The behavior of the model circuit resembles several reported characteristics of cortical circuits, such as a delay of several milliseconds between excitation and inhibition and a stronger synchronization amongst the local interneuron population than amongst the pyramidal cell population (Hasenstaub et al., 2005; Atallah and Scanziani, 2009).

In line with previous results that were obtained using similar circuits, the level of synchronization and the oscillation frequency were found to depend on the ratio of depolarization between the cell types (Buia and Tiesinga, 2006). The oscillation frequency of the circuit's activity increased with an increase in the depolarization of both pyramidal cells and interneurons, i.e., along the diagonal in Figure 1C (see also Figure 1D). The circuit generated oscillations with frequencies in the beta and gamma ranges (20–90 Hz). Synchrony within the circuit was quantified by the pairwise phase consistency (PPC), an unbiased measure that compares the phases of the spike density trace (see Figure 1B for an example) at which spikes occur. When all spikes occur at the same time in every period of the oscillation, their corresponding phases will be the same and the phase consistency will be 1, while if spikes occur at random times, their phase distribution will approach uniformity and the PPC is 0 (see Methods). By increasing the depolarization of one cell type in the circuit, but decreasing that of the other type (line E in Figure 1), synchronization could be varied between its peak value and the onset of asynchrony. Note that asynchrony does not mean that the network is silent. In fact, in the light gray area between the colored PING area and the dark gray ING, both cell types are spiking at physiologically relevant firing rates (see Figure SI2 E,F).

The clear relation between depolarization of the neuron types in the circuit on the one hand and frequency and synchrony of the circuit on the other hand, allows us to study the effect of synchrony and frequency in the context of communication between circuits.

### Synchronization between areas connected with unidirectional projections occurs along a tilted arnold tongue

Communication between brain areas is mediated by long-range synaptic connections that are generally thought to be predominantly excitatory (Douglas and Martin, 2004; Stepanyants et al., 2009; Harris and Shepherd, 2015), but see (Caputi et al., 2013). Synchronization between two intrinsically oscillating circuits is expected to depend on the absolute strength of the synaptic connections between the circuits, the ratio of feedforward vs. feedback connections and the number of projections to downstream pyramidal cells and interneurons as well as the difference in latency between the projections (Tiesinga and Sejnowski, 2009). Here, we study the synchronization in a unidirectional network of two oscillating circuits (Figure 2A) for a range of synaptic strengths. The inter-circuit projections were purely excitatory and targeted both pyramidal cells and interneurons in the receiving circuit with the same total synaptic conductance (the product of the number of synapses per neuron and their unitary strength). The depolarizing current the receiver circuit received was kept fixed such that, in isolation, it oscillated at ~43 Hz and had an intermediate level of synchrony. The depolarization of the sending circuit was varied such that its frequency increased from 29 to 75 Hz, while keeping its synchrony at an intermediate level. The synchronization between the circuits was determined as the coherence between the respective spike density traces, taken at the frequency of the sender (Figure 2B). The coherence decreased when the frequency difference between the circuits increased (compare Figure 2B and Figure SI5A).

Synchronization between two unidirectionally coupled circuits occurred when the oscillation frequency of the sending circuit was higher than that of the receiving circuit and the connections were strong enough to sufficiently increase the receiving circuit's frequency to match that of the sending circuit (Figure 2B). The network's behavior shows similarities with that of a simple non-linear oscillator, such as a pendulum, driven by an oscillatory input (Pikovsky et al., 2001). For the non-linear oscillator, synchronization is found when the input's frequency lies within a small frequency interval around the intrinsic frequency of the oscillator. The width of this interval increases with the amplitude of the drive, yielding a triangular area of synchronization in the amplitude-frequency plot and is usually referred to as an Arnold tongue (Pikovsky et al., 2001; Tiesinga, 2002). In agreement with the observations of a non-linear oscillator, the network model shows an expansion of the range of frequencies that lead to synchronization with connection strength. However, unlike the non-linear oscillator, for the model network the area of synchrony is asymmetric around the intrinsic frequency of the receiver (Figure 2B). The asymmetry can be understood by considering that increased synaptic strength not only increases the size of input modulation to the receiver, but also increases its average input, because it is excitatory. To study this effect we considered an isolated model circuit and drove it with a sinusoid input, for which the average and amplitude could be modulated independently. The simple model showed that increasing the amplitude of the input only yielded a conventionally shaped Arnold tongue (see Figure 2C, left), while increasing the average input caused an increase in the intrinsic frequency of the circuit, creating a tilted axis for synchronization (Figure 2C, middle). Taken together, modulation of both amplitude and average input explains the finding that synaptically connected circuits show synchronization in a rightward tilted Arnold tongue (compare Figure 2C, right and Figure 2B).

In the center of the Arnold tongue, the driving frequency and the intrinsic frequency of the receiving circuit were identical and the receiver showed resonance (i.e., high PPC, see Figure SI4C and the black contours in Figure 2). However, resonance was not required to achieve high synchronization. For all conditions leading to high coherence (>0.90) and hence similar oscillation frequencies for the two circuits, the average phase difference between the circuits could be determined using the spike density traces (Figure 2D). In all cases, circuit 2 lagged circuit 1, but the phase difference varied with the difference in intrinsic frequencies between the circuit (this direction is perpendicular to the axis of the Arnold tongue in Figure 2B), in agreement with expectations based on the non-linear oscillator case (Pikovsky et al., 2001). Small phase differences were found for small intrinsic frequency differences. For increasing intrinsic frequency differences, excitatory volleys from the sender overlapped more and more with the period of inhibition of the previous oscillation cycle of the lower-frequency receiving circuit, therefore delaying the entrainment by the sender, leading to higher phase differences of up to $\frac{8}{5}\pi$, that is 0.8 of a period. For the conditions presented in Figure 2D, with an axonal delay of 5 ms between the circuits and an observed intrinsic circuit response time of ~3 ms, a 73 Hz oscillation frequency resulted in a resonance phase difference around the axis of the Arnold tongue of about $\frac{73\times \left(5\;+\;3\right)}{1000}=0.58$ period, or ≈ 1.15π, as can be seen in Figure 2D.

The spread of the Arnold tongue around the area of resonance was not only determined by the total synaptic conductance from circuit 1 to circuit 2, but also depended on the ratio of excitation and inhibition recruited by the projection to circuit 2 (Figure SI5). When intercircuit projections recruited more excitation, the Arnold tongue narrowed, though the same phase difference range was achieved, and spikes in the receiving circuit were better aligned to the activity in the sending circuit (i.e., high PPC). Increase of inhibition led to a widening of the Arnold tongue, but again the phase difference range was unchanged across the range of the tongue. In addition, there was a decrease in PPC in circuit 2, both relative to the spike density of circuit 1 and that of circuit 2. This indicates that feedforward projections that preferably recruit excitation facilitate strong synchronization in a narrow range of intrinsic frequency differences between the circuits. This makes the degree of the synchronization and phase difference more sensitive to small changes in the intrinsic frequency difference.

In summary, in a unidirectionally coupled network of two oscillating circuits, synchronization occurred along a tilted Arnold tongue. For the connection settings used here, synchrony was thus only achieved for driving frequencies that are above the intrinsic frequency of the receiving circuit. It therefore seems advantageous for receiving circuits to oscillate at low intrinsic frequencies, as this can be used to dynamically create a feedforward pathway. On the Arnold tongue the phase difference between the circuits increased from small to large when the sender frequency increases from that of the receiver to above that of the receiver. Since the phase relation is determined by the intrinsic frequency difference, which in turn is determined by the static depolarizing currents administered to the circuits, this network setup allows the phase relation between the circuits to be completely controlled by the level of excitation in the circuits. In the brain, feedforward and feedback signals can therefore change the synchronization between circuits, but also their phase difference, by targeting one or both of the circuits in the network, without having to explicitly impose external oscillations or phase relations to the circuits. In the next sections, we will utilize the intrinsic frequency difference between the sender and receiver to study the importance of phase relations in information transfer in a network.

### Oscillations create windows of high input susceptibility

The functional relevance of oscillatory activity on coding and transmission of information has been extensively discussed in recent years, but it has proven difficult to test it directly in experiments. In the formulation of the Communication Through Coherence hypothesis (see Fries, 2005, 2015), as well as in related hypotheses (such as in Akam and Kullmann, 2014), it was recognized that oscillatory activity of neural circuits can generate periodic time windows of high input susceptibility (Tiesinga et al., 2004). High susceptibility can arise due to a simultaneous increase in depolarization of the neurons in the circuit, either by decreased inhibition or increased excitation, shifting the spike threshold to lower input strengths. Also, oscillations have been proposed to separate inputs in time based on their strengths, with strong inputs leading to earlier firing than weaker ones (McLelland and Paulsen, 2009). Earlier events are subsequently more likely to cause downstream effects than later events, as those later events will be masked by local inhibition recruited by the preceding inputs (Atallah and Scanziani, 2009).

To test for susceptibility windows due to oscillatory activity in our model, we applied pulsed inputs to a fraction of the pyramidal cells in circuit 1 (Figure 3A). Pulses resembled EPSCs caused by a single presynaptic action potential and were applied to all receiving cells simultaneously. These pulses could be viewed as a coherent external input from another cortical area, or could be considered the result of a brief light pulse when the neurons are expressing channelrhodopsin and are therefore light sensitive. The simulations were repeated without pulse application so that the network's activity after pulse application could be compared to the same condition without pulse. The timing of the pulse relative to the ongoing oscillation, indicated by θ, was varied from 0 (at the peak) to 2π (at the peak of the next period, see Figure 3A).

We analyzed the effects of pulse application on the circuit's spike rate and on the oscillatory activity. Pulse application induced both transient and persistent changes in the activity of the local circuit. To capture the transient effects of a pulse, we compared the circuit's behavior in the first oscillation period after pulse onset to the same circuit's behavior without pulse (Figure 3C). Pulsed inputs significantly increased the firing rate of the circuit in the first oscillation period after the pulse (Figure 3C, left). However, the size of the effect depended on the timing of the pulse: The effect was strongest for pulses applied around the peak (0 or 2π) of the oscillation (significant circular-to-linear correlation, with ρ = 0.83 and *p* < 0.0001).

Furthermore, pulses changed the oscillatory characteristics of the circuit that received the pulse. After a pulse, the peak of the next excitatory volley shifted forward, reducing the time of the next peak in excitation (negative values in Figure 3C, middle), with the biggest changes occurring when the pulse arrived between two excitatory volleys (ρ = 0.77 and *p* < 0.0001). In other words, pulses always shorten the oscillation period and hence the circuit has a Type I (all advancing) phase response curve (PRC; Hansel et al., 1995). In addition, the height of the peak of the next excitatory volley increased or decreased, indicating that the pulse synchronized, respectively desynchronized, the spikes of the circuit (Figure 3C, right), with synchronization being strongest for pulses arriving before the peak of the excitatory volley (ρ = 0.68 and *p* < 0.0001).

To assess the long-term effects of pulsed inputs, we developed continuous, time-resolved measures capturing the same three spike density characteristics as before: Spike rate-, peak time-, and peak height difference. The equivalent of the spike rate per period after the pulse was the period-averaged and smoothed number of spikes, the peak time was captured by the instantaneous frequency and the peak height by the instantaneous power of the spike density trace. In the first period after pulse onset, these measures yielded the same results as in Figure 3C (Figure SI8). In agreement with previous results (Tiesinga and Sejnowski, 2010), the frequency increase was not followed by a period of reduced frequency, indicating that the induced phase shift in the local circuit was persistent.

These findings not only demonstrate that the oscillation induced susceptibility windows, but also indicate that these windows can be specific to signal feature. The three signal features studied, firing rate, frequency, and power, all show a phase specific response, but their preferred phases (at which the change is maximal) do not coincide, allowing for temporal multiplexing of outputs.

The presence of input susceptibility windows potentially affects communication between circuits, as the overlap of the susceptibility windows of the two circuits depends on the phase difference between the circuits. As described in the previous section, for the conditions used in these simulations, pulses had the same arrival phase in the two circuits when the phase difference between the circuits was slightly above π, which reflected the contributions of the synaptic delay, intrinsic dynamics and oscillation frequency of the circuits. One might therefore expect the optimal phase difference for transmission of a pulse applied to circuit 1 to be equal for all three analyzed features and to be, for the condition studied, around a phase difference of π. However, processing and coding occurring in circuit 1 might change the nature of the signal significantly and therefore alter the requirements for optimal susceptibility of the receiving circuit 2.

To test the importance of matching susceptibility windows directly, we varied the phase differences between the circuits by changing the intrinsic frequency of the receiver, while applying pulses to the sender (Figure 3B). Changes in spike rate, excitatory peak time and -height in circuit 2 after the pulse are represented twice in Figure 3D, once against the pulse arrival phase relative to circuit 1 (θ_1_, top row) and once against the pulse arrival phase relative to circuit 2 (θ_2_, bottom row).

Like in circuit 1, the spike rate in circuit 1 increased in the period after the pulse. The curve of the spike rate change in circuit 2 shifted with phase difference against pulse phase in circuit 1, but not when expressed relative to the phase in circuit 2. In addition, the maximal spike rate change was modulated with phase difference (significant correlation, ρ = 0.49, see also Figure SI7 and Table SI3), indicating that phase difference affected transfer of spike information to circuit 2. As expected, optimal transfer of spike rate change was found around Δφ = π. Like spike rate change, the amplitude of the peak height change depended on phase difference (significant correlation for both maximum and minimum, see Figure SI7 and Table SI3) and the curve shifted with phase difference relative to θ_1_. However, peak timing did not show a phase difference dependence (ρ = 0.16, *p* = 0.23). Instead, the change in peak time only depended on the pulse arrival phase in circuit 1, indicating that in a condition where a receiving circuit is coherent with a sending circuit, the receiver will follow the phase shifts of the sender.

The presented findings suggest that oscillations introduce input susceptibility windows, i.e., windows in which inputs are effectively translated to neural activity patterns, which are followed by windows in which translation is ineffective. These windows affect communication of the signals to downstream circuits, since phase differences between the circuits affect the overlap of the susceptibility windows of the two circuits. These phase differences affect transfer of oscillatory power and firing rate information between circuits, but not frequency.

### Synchrony and good phase relations lead to high information transfer

The phase dependent input-output relationship found for pulsed inputs is expected to affect information transfer between circuits as described by the Communication Through Coherence hypothesis. To directly test this in our model, we adapted the input protocol: On top of the static depolarizing current, the pyramidal cells in each circuit also received colored noise currents, which were identical for all pyramids in a given circuit, but were different and uncorrelated (independent) between circuits, thereby mimicking inputs from two different sources of information (Figure 4A). As for the pulses, the noise current induced changes in the spike density, frequency, and power of the oscillation, as well as in the population firing rates of the two circuits. The intrinsic frequency of the sending circuit and the connection strength between the circuits were varied, leading to a range of synchrony and phase conditions. For each condition, the “information transfer” between the circuits was determined as the correlation coefficient of each of the three signal traces, i.e., instantaneous frequency, power, and firing rate per oscillation period. These signals are itself not oscillatory, allowing for a comparison of the activity of the circuits without being confounded by the shared periodic activity. Interpreted within the approximation of linear information transfer, wherein the response of the second circuit contains an instantaneously rescaled component due to the signal from circuit 1, the correlation measures the size of this component. This approach can be used because the signals are dictated by the independent, uncorrelated noise inputs and are normally distributed. A correlation coefficient of 1 indicated that all variability in circuit 2 was explained by variability in circuit 1 and hence indicated “good” communication, while a correlation coefficient not significantly different from 0 indicated that the variability in the two circuits was uncorrelated and was therefore not caused by the projection between the circuits.

For all signal types, i.e., frequency, power, and firing rate, correlations increased with coherence between the circuits (Figures 4B–D left). For frequency, the increased correlation was fully explained by the coherence between the circuits, while for power and firing rate, high coherence led to both high and low levels of correlation. In addition to high levels of coherence (i.e., frequency matching), these signals also needed particular phase relations between the circuits to be transferred well (Figures 4C,D right). Correlations were lost for all coherence levels and phases when the traces of circuit 2 were shuffled in time (Figure SI9A), i.e., when breaking the temporal structure in the data. To test whether this temporal structure was dominated by the structure in oscillation periods, we divided the traces of circuit 2 into windows of one oscillation period and randomly shuffled the periods before correlation analysis. This procedure maintained the temporal structure within oscillation periods. Period-shuffling reduced correlation coefficients to around 0 (Figure SI9B), indicating that the correlation coefficients reported in Figure 4 were not caused by a temporal structure that is shared by the oscillation periods.

Similar results to Figure 4 were obtained when, for the same data, the mutual information between the circuits was computed (Figure SI10), however, this method requires more data to yield adequate estimates. Variability between simulation runs was high for the power signal, but not frequency and firing rate (Figure SI11) and considering individual runs in those cases led to the same conclusions.

The optimal phase of information transfer for the reported network was 1.15π, which coincided with the phase difference leading to highest PPC_2 → 1_, as reported in the section “Synchronization between areas connected with unidirectional projections occurs along a tilted Arnold tongue”. As postulated in that section, this optimum was an emergent property of the network and depends on the oscillation frequency and synaptic delay. When inter-circuit synaptic delay or the oscillation frequency decreased, the optimal phase difference shifted to lower values, while the level of information transfer at the optimal phase remained unchanged (Figures 5A,B). In addition to synaptic delay and oscillation frequency, the optimal phase depended on the ratio of the inhibition and excitation recruited by the projections from the sending to the receiving circuit. When the intercircuit E → I conductance was low, as was discussed in the section “Synchronization between areas connected with unidirectional projections occurs along a tilted Arnold tongue” and Figure SI5, the circuits synchronized only within a smaller range of intrinsic frequency differences (narrow Arnold tongue), while the level of synchronization was higher. Figure 5C shows that this high level of synchronization also led to a high level of information transfer. A stronger activation of inhibition by the feedforward projection broadened the range of frequency differences at which the circuits synchronize (Figure SI5), but led to a lower level of information transfer. These data indicate that there is a trade-off between the ease of synchronization and the level of communication that can be obtained between neural circuits: Easy synchronization means a low level of communication, while synchronization in a narrow frequency band leads to good communication.

### Phase relations allow for input selection

Coherence- and phase-dependent communication as shown in the previous section potentially provides a mechanism for input selection between local circuits in different brain areas, by allowing a receiver to dynamically synchronize to one sender at the exclusion of other senders. We tested this mechanism in a feedforward network with two sending circuit (1a and 1b) projecting to the same receiver (2, Figure 6A). Figure 6 shows one example simulation run. By varying the depolarization (Figure 6B) and therefore the intrinsic frequency (Figure 6C) of sender 1a and the receiver in three epochs of a single trial, we achieved the following three epochs (Figures 6D,E): (1) high coherence between 1b and 2 at 55 Hz, with a 0.7π phase difference; (2) high coherence between 1a and 2 at 73 Hz, and a 1.2π phase difference; (3) high coherence between 1a and 2 at 73 Hz, at a 0.8π phase difference.

In line with the results from the previous section, the correlation coefficient for LFP frequency followed the changes in coherence between the sender and receiver (Figure 6F top). While sender 1b had a high correlation coefficient with circuit 2 in the first epoch, it dropped and remained low in epoch 2 and 3. Sender 1a had a low correlation coefficient for epoch 1, but followed the increase in coherence and had a high correlation coefficient in epochs 2 and 3. Power and firing rate also followed the coherence, but, as before, the outcome depended on phase difference. While the correlation coefficient of sender 1a and the receiver was high in the second epoch for both power and firing rate, the third epoch showed a divergence between the two signals. Information transfer in the power signal went up in epoch 3, as the phase difference decreased. At the same time, the correlation coefficient of the firing rate between 1a and 2 went down with the decrease in phase difference. Those findings are in agreement with the findings presented in (Figures 4C,D), where it was shown that the peak of power transfer is at lower phase differences than the peak of firing rate transfer.

These findings suggest that the synchrony and phase related requirements for communication can effectively switch the direction of communication on short time scales and in single trials.

In a state where each of the sending circuits are equivalent in terms of frequency and amplitude (synchrony), a downstream circuit will switch between the information streams of the senders on a time scale of several tens to a few 100 ms. From our previous findings, we hypothesize that communication from one of the senders can be stabilized over longer time scales in two ways: (1) by increasing the intrinsic frequency of the relevant sender; (2) by decreasing the intrinsic frequency of the other (irrelevant) sender(s) in the network. The two cases have opposing effects on the phase difference between the sender of interest and receiver: (1) leads to an increase in phase difference, while (2) leads to a decrease. The resulting change in information transfer is therefore expected to depend on the optimal phase difference and hence on the oscillation frequency and axonal delay between the circuits.

We tested these predictions for a network with an optimal phase difference of around 0.8π. When the frequency of circuit 1a was increased, the coherence between circuit 1a and 2 increased, as did their phase difference (Figure SI11). Reducing the frequency of circuit 1a instead led to an increase of coherence between 1b and 2, while their phase relation remained stable. This experiment was repeated for a range of synaptic strengths, to allow for different phase relations between the circuits. As expected, phase relations around 0.8π led to the highest information transfer between the circuits.

## Discussion

In summary, we showed that two brain circuits, both showing oscillatory activity emerging from local circuit activation according to the PING mechanism, synchronized their activity across a range of frequency differences, levels of internal synchronization and I/E-ratios of the projections. The frequency at which synchronization occurred was determined by the sending circuit, while the level of coherence and the phase difference was set by the frequency difference between the two intrinsic oscillations. We showed that the presence of oscillatory activity affected the susceptibility of the circuits to inputs, creating alternating windows of low and high input-output gain. Coherence and phase difference between the circuits directly affected the information transfer in the network by aligning these susceptibility windows. When the circuits had the same oscillation frequency (i.e., strong synchronization) and the phase difference between them corresponded to the synaptic delay and the time needed to integrate information in the receiving circuit, information transfer was optimized. We showed that in the model, changes in synchrony and phase difference could be used to set up or end information transfer in a network of two senders and one receiving circuit, either by changing the drive to the sender or receiver, or by changing the relative recruitment of inhibition in the receiving circuit.

Studies into dynamic communication, often in the context of attention or goal-directed tasks, have given us a wide range of paradigms in which pair-wise synchrony in the gamma band correlates with task relevance (Gregoriou et al., 2009; Buschman et al., 2012; Roberts et al., 2013; Saalmann et al., 2012 and many others). In two attention studies, the hypothesis that synchrony increases with attention was directly tested by recording from two sites in V1 that projected to the same site in V4 (Bosman et al., 2012; Grothe et al., 2012). In both studies, the attended V1 receptive field had a stronger gamma synchronization to the V4 site than the non-attended site and there was a causal influence in the direction of information transfer from V1 to V4 at the peak gamma frequency (Bosman et al., 2012). Our model aligns with these results and shows that these task-related changes in synchrony: (1) can be caused by changes in the activity of either the sender or the receiving circuits in the network and do not require anatomical (synaptic plasticity) or physiological (excitability) changes and do not require a common input or external oscillator; (2) can directly reflect or cause task-relevant changes in communication between brain areas involved in the task.

In addition, our PING model predicts coherence in a feedforward network can only be achieved if there is a frequency hierarchy amongst the circuits in the network, with receivers having lower frequencies than senders. This is in agreement with the experimental finding from Bosman et al. (2012), showing that V1 areas processing attended stimuli increased their oscillation frequency, but not power, in the gamma band. For this model, it was previously demonstrated that changes in frequency of the sender, for instance caused by stimulus features such as contrast, do not affect the synchrony between the sending and receiving circuits (Roberts et al., 2013). Yet, communication can be broken by giving inputs that either increase the frequency of the sender beyond that of the receiver, or that affects the phase relation between the circuits. These characteristics of gamma band synchronization make it a potentially suitable mediator of bottom-up dynamic communication, as it sets a clear direction of communication, which is not easily broken by fluctuations in input, but can be dynamically set up and broken down.

**Figure 7:**
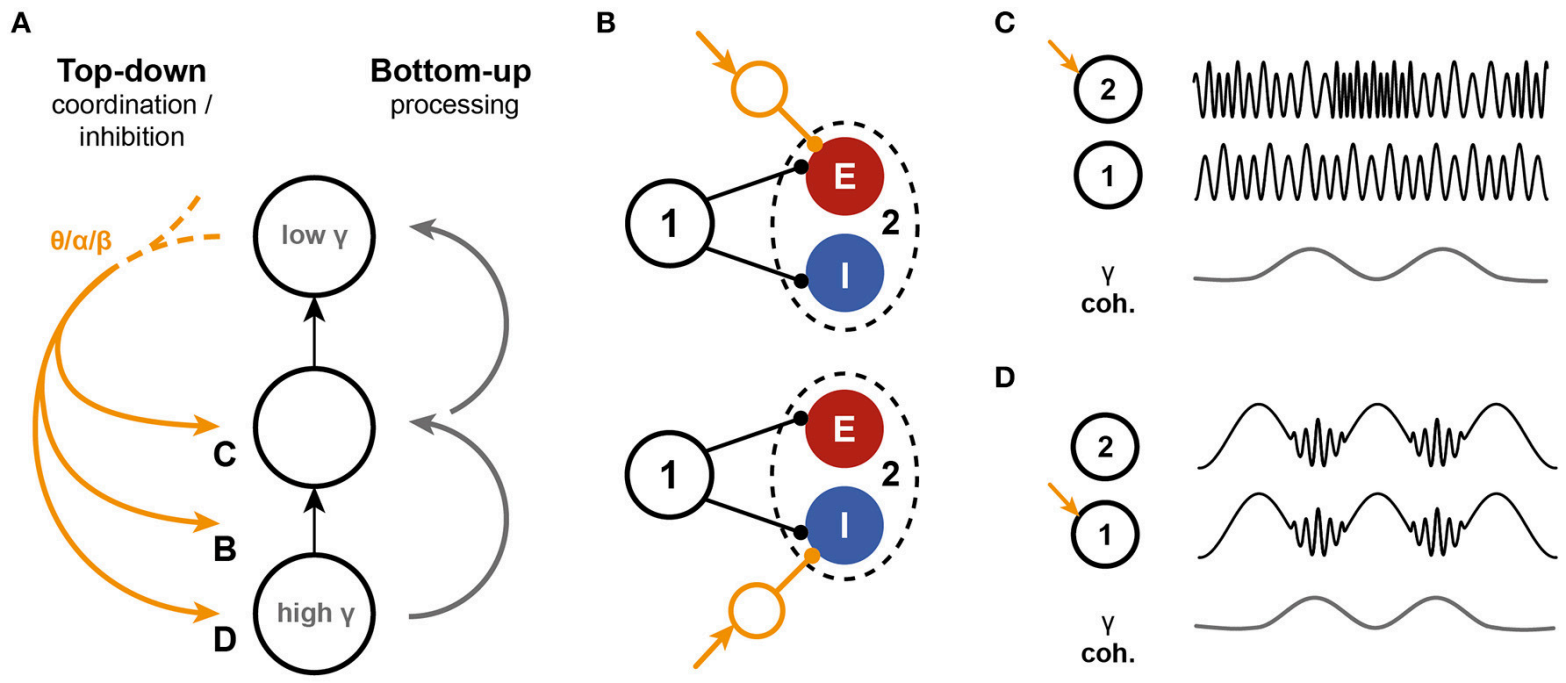
**Interactions between top-down inputs and the PING model**. **(A)** Bottom-up processing is associated with gamma oscillations. The model presented here predicts a decrease in intrinsic oscillation frequency along the hierarchy in the network. On the other hand, top-down inputs are mediated through low frequencies. These slow oscillations can interact with the bottom-up information stream in several different ways. **(B)** The model predicts that the ratio of excitation to inhibition that is recruited by a bottom-up stream affects the information transfer, by altering the phase locking of the receiver spikes. Top-down input can interfere by shifting the balance to excitation of inhibition. **(C,D)** Slow oscillations can also directly target the oscillations in either the sender **(C)**, the receiver **(D)** or both local circuits. The effects of such interference need further investigation.

To our knowledge, the feedforward gamma frequency hierarchy prediction has not been directly tested experimentally. On the other hand, evidence is building up for a division of labor between frequency bands (Figure 7A), with gamma frequencies being related to feedforward information transfer, while feedback has been linked to the much lower alpha and/or beta frequency bands (Bastos et al., 2014; van Kerkoerle et al., 2014; Michalareas et al., 2016 and see Bressler and Richter, 2015 for a review) and theta-gamma coordination (Landau et al., 2015; Voloh et al., 2015). These feedback signals can (1) facilitate synchrony and communication (Saalmann et al., 2012); (2) coordinate feedforward communication, for instance to allow sampling of the input space (Lisman and Jensen, 2013); or (3) inhibit it locally (Jensen and Mazaheri, 2010). The mechanisms that underlie these different modes of feedback through slow rhythms onto the bottom-up processing of stimuli are largely unknown. Based on the presented model, several interactions are feasible, summarized in Figure 7. First, feedback could affect the susceptibility of receivers to inputs by recruiting local circuits (Figure 7B), either through feedforward excitation or -inhibition, creating an input gate. Second, feedback could interact directly with the gamma oscillation by targeting either the sender (Figure 7C) or the receiver (Figure 7D) or both (Quax and Tiesinga, 2015). Additional work is needed to assess the feasibility of these potential mechanisms and their impact on coherence, power, spike rate, and other measures that can be directly assessed in experiments.

Similarly, the impact of lateral or recurrent excitation and inhibition between circuits needs to be assessed. These connections are highly abundant in V1, where they are thought to mediate zero-phase lag synchronization between circuits (Vinck and Bosman, 2016), a regime that cannot be achieved in the feedforward network setup presented here. In Vinck and Bosman (2016), the authors predict that gamma synchronization between distributed circuits in V1 reflects the extent to which these circuits predict each other's information. Unlike the competition shown in Figure 6, this shared oscillatory signature could strengthen the integration of the information from these distributed locations, since the circuits have similar frequency and phase relations with downstream areas. An extension of the model is needed to test this hypothesis directly.

More so than for the relation between coherence and task relevant communication, the significance of phase relations in dynamic communication has been harder to assess directly in experiments. In agreement with our model, particular phase relations were previously linked to high correlation between LFPs from different locations in the brain (Womelsdorf et al., 2007) and phase relations were found to be similar for functionally linked neurons and circuits (Canolty et al., 2010). A study in macaque visual cortex indicated that the phase relation, but not the phase itself, carried information about the presented stimulus (Besserve et al., 2015). More controlled studies into these potential coding schemes are needed. These findings were replicated by modeling studies, showing that the phase difference affected the routing of information (Battaglia et al., 2012) and the amount of information transferred (Buehlmann and Deco, 2010). Unlike in previously reported network models, where phase relation were found to be mostly restricted to in- or out-of-phase (Barardi et al., 2014), or phase had to be set by adjusting the synaptic delay (Buehlmann and Deco, 2010), we show that the phase relation in a unidirectional network can take virtually any value, by adjusting the frequency difference in the network. The relation between the phase difference in a unidirectional network and the difference between driving frequency and intrinsic frequency of the receiver was shown previously at the single neuron level (Tiesinga and Sejnowski, 2009) and was confirmed experimentally, in hippocampal slices, where CA3 neurons were optogenetically modulated by an oscillatory drive (Akam et al., 2012). As in our model, phase difference increased when the driving frequency increased relative to the intrinsic frequency. This approach allowed us to directly address the possibility of phase-mediated gating of information transfer in our two circuit network (Fries, 2005, 2015) and gives a potential mechanism for dynamic control of phase relations in the brain.

Phase-resetting is one of the signatures of dynamic phase relations between neurons or circuits and has been linked to attention (see Voloh and Womelsdorf, 2016 for a review). Our findings suggest that phase changes can be caused by the onset of (changes in) input to either the sending or receiving circuits, and last as long as the input. It was previously shown that advancing phase shifts can be reliably induced in isolated PING circuits by applying pulsed inputs to the interneuron population (Tiesinga and Sejnowski, 2010). Here, we contributed that pulses to the pyramidal cell population also induce advancing phase shifts, though the maximum shift is lower than for pulses to the interneuron population. Phase shifts induced in the sending circuit of a feedforward network were transferred to the receiver, and hence did result in effective phase-resetting in the network, but did not create a change in phase difference between the circuits. A pulse applied to the receiver circuit led to a short-lived change in phase difference, as the phase difference was restored to its original value in the next oscillation periods (data not shown). We did not assess the effect of pulses under different levels of coupling between the circuits and for more complex network architectures. The potential sources in phase resetting and phase shifting remain to be explored further in future work.

Like the PING mechanism, the ING mechanism has been implicated in task-relevant modulation of visual attention (Vinck et al., 2013). The characteristics of the ING mechanism differ from the PING mechanism in several ways. Pyramidal cell spiking activity is generally much lower and pyramidal cell firing, though concentrated in the trough of inhibition, is less structured than in the PING mechanism, where pyramidal cell firing peaks a few milliseconds before interneuron firing (Tiesinga and Sejnowski, 2009; see also Figure SI13). These characteristics are likely to affect the synchronization amongst connected ING circuits: The low and imprecise firing renders excitatory projections between circuits less effective as synchronizers and excitatory projections are expected to only allow synchronization at specific frequency relations between the circuits. Instead, synchronization between ING circuits is expected to be best achieved through direct inhibitory connections between the circuits. Synchronization through direct inhibitory connections are expected to result in markedly different network dynamics: (1) Inhibitory connections allow for synchronization at both higher and lower frequencies of the receiver and hence do not require a frequency hierarchy; (2) Inhibitory connections collapse the possible phase relations between the two circuits to a small range of values (see Figure SI14). The effects on information coding and transfer in ING circuits require additional work. The marked differences between the ING and PING mechanisms speak to a division of functions between the two circuit motifs. For example, while the ING motif could function as a fast all-or-nothing synchronizer, the PING motif would be the prime candidate for setting up more complex, multi-level networks for information routing. This would be in line with previous results, in which ING was implicated to be the dominant mechanism during the attention cue period (Vinck et al., 2013), while pyramidal cell locking related to the PING mechanism was found during visual stimulation (Vinck et al., 2013; Perrenoud et al., 2016). The characteristics of ING-mediated information transfer, as well as the possible functional differences of ING and PING, remain to be elucidated.

The experimental link between oscillatory activity and task dynamics shows a (causal) relation between dynamic communication and a synchrony structure, but do not clarify the nature of this relation: Are oscillations the infrastructure for task-relevant communication, or do they dynamically encode the task-relevant information? The assumption underlying the CTC-hypothesis is that oscillations affect the susceptibility to input (Fries, 2015), as the synchronized release of inhibition and subsequent synchronized rise of excitation, have been shown to promote spike generation (Azouz and Gray, 2003). Modulation of spiking activity in a downstream area related to oscillatory activity in a sender has been shown in several experiments (Canolty et al., 2010; Jia et al., 2013; Zandvakili and Kohn, 2015). In the model, spiking activity in the downstream circuit was locked most strongly (high PPC_1 → 2_) along the axis of the Arnold tongue. At those phase differences, the excitatory volley of the sending circuit arrived around the time of the excitatory volley of the receiving circuit. This timing is optimal: In Figure 3 we showed that inputs were converted to firing rate most effectively when the input coincided with the excitatory volley in the circuit. In between volleys, when the PING-mechanism is dominated by inhibition (Tiesinga and Sejnowski, 2009), pulses still led to increases in firing rate, but the effects were smaller.

We showed that not only firing rate is affected by the phase of the input, but changes in oscillation frequency itself and power at this frequency were similarly affected by phase of input. In contrast to firing rate, the timing of the next volley (frequency change) was most effected by pulses in between two volleys, i.e., when the excitatory cells in the PING mechanism are about to overcome the inhibitory volley, in agreement with previous results (Tiesinga and Sejnowski, 2010). Peak height was affected most at the rising phase of the excitatory volley. As a result, for the three coding schemes we studied information transfer depended on the intercircuit phase difference, but had different optimal phase difference. This potentially allows for phase-dependent multiplexing of the incoming signal. In this context, oscillations could not only be mediators of effective communication, but could also be potential information carriers, as was recently reported in Watrous et al. (2015). The picture diversifies further when we consider recent evidence (Dann et al., 2016) indicating that the cortex might be organized in a more task-distributed way: Some neurons are densely connected, and synchronized to ongoing oscillations, while others project more locally, and are less coupled to ongoing oscillations. The effect of this potential separation of cell populations on coding, communication and their interaction is unclear.

These different lines of evidence indicate that the presence of PING creates (1) susceptibility windows and that (2) these windows are different for the various features of the PING-circuit. This allows for multiplexing of information into different communication streams, each with its own characteristics. Whether these different features are decoded and interpreted by receiving cells or circuits in the brain, and if so, to what extent, remains a topic of debate. However, even if gamma oscillations and the described windowing and multiplexing features are mere consequences of the underlying anatomy and activity and bear no functional meaning, it is conceivable that understanding them can give us important insights into the origin of the electrophysiological signals that are recorded (Womelsdorf et al., 2014), as we can potentially trace them back to a oscillatory mechanism (PING) and identify the timing of the input.

Summarizing, the results we presented here indicate that a coherence-based structure can underlie effective communication in a two-circuit network with excitatory intercircuit connections. We find that not only synchrony, but a combination of high synchronization and a specific phase relation is required for optimal information transfer in the network, giving rise to highly dynamic communication structure.

## Author contributions

MtW and PT designed the experiment. MtW implemented the model, and collected and analyzed the data with input from PT. MtW and PT wrote the manuscript.

## Funding

This work is supported by NeuroSeeker, a project funded by the European Union's Seventh Framework Programme (FP7/2007-2013) under grant agreement nr. 600925.

### Conflict of interest statement

The authors declare that the research was conducted in the absence of any commercial or financial relationships that could be construed as a potential conflict of interest.
