# Supplementary material for: Phase Difference between Model Cortical Areas Determines Level of Information Transfer
Source: Front Comput Neurosci. 2017 Feb 9;11:6. doi: 10.3389/fncom.2017.00006 (PMC5298997; doi:10.3389/fncom.2017.00006)
Supplement: Supplementary file 1 [file DataSheet1.PDF]

Supplementary Material

Phase between model cortical areas determines information transfer

Marije ter Wal*, Paul H Tiesinga

*** Correspondence:** Marije ter Wal: m.terwal@donders.ru.nl

1 Models

1.1 Neuron models

1.1.1 Interneurons

A single area contains 100 inhibitory, fast-spiking interneurons. The membrane potential V_i (mV) of these interneurons obeys:

$$C_m \frac{dV_i}{dt} = -I_{\mathrm{Na}} - I_{\mathrm{K}} - I_{\mathrm{L}} - I_{\mathrm{GABA}} - I_{\mathrm{AMPA}} + I_{\mathrm{Inj}} + C_m\xi \tag{1}$$

The right hand side terms represent, respectively, the sodium and potassium currents, a leak current and synaptic inputs (Wang & Buzsáki, 1996). C_m is the membrane capacitance (μF/cm^2) and $C_m\xi$ is a noise term. I_{Inj} represents all external input currents into the cell. The currents in (1) are modeled as:

$$\begin{aligned}
I_{\mathrm{L}} &= g_{\mathrm{L}}(V_i - E_{\mathrm{L}})\\
I_{\mathrm{Na}} &= g_{\mathrm{Na}} m_\infty^3(V_i) h (V_i - E_{\mathrm{Na}})\\
I_{\mathrm{K}} &= g_{\mathrm{K}} n^4 (V_i - E_{\mathrm{K}})\\
I_{\mathrm{GABA}} &= g_{ii} s_{ii}^{\mathrm{tot}}(t)(V_i - E_{\mathrm{GABA}})\\
I_{\mathrm{AMPA}} &= g_{ei} s_{ei}^{\mathrm{tot}}(t)(V_i - E_{\mathrm{AMPA}})
\end{aligned} \tag{2}$$

Where m, h, n and s are gating variables. Gating variable m has fast dynamics and has been replaced by its asymptotic value m_∞. The dynamics of h and n are described by differential equations:

$$\begin{aligned}
m_\infty &= \frac{\alpha_m}{\alpha_m + \beta_m}\\
\frac{dh}{dt} &= \phi\big(\alpha_h(1-h) - \beta_h h\big)\\
\frac{dn}{dt} &= \phi\big(\alpha_n(1-n) - \beta_n n\big)
\end{aligned} \tag{3}$$

With rate constants:

$$\begin{aligned}
\alpha_m &= \frac{-0.1(V+35)}{\exp(-0.1(V+35)) - 1}\\
\beta_m &= 4\exp\left(-\frac{V+60}{18}\right)\\
\alpha_h &= 0.07\exp\left(-\frac{V+58}{20}\right)
\end{aligned} \tag{4}$$

$\beta_h = \left[\exp\left(-0.1(V+28)\right)+1\right]^{-1}$
$\alpha_n = \frac{-0.01(V+34)}{\exp\left(-0.1(V+34)\right)-1}$
$\beta_n = 0.125\exp\left(-\frac{V+44}{80}\right)$

The parameter values used in the model can be found in Table SI1. An example membrane potential trace and the fI-curve of these interneurons are shown in Figure SI2.

1.1.2 Pyramidal cells

The model for the 400 pyramidal cells in the local circuit is slightly different from that of the interneurons described above (Buia & Tiesinga, 2006; Golomb & Amitai, 1997). It incorporates a sodium current, persistent sodium current, delayed rectifier potassium current and the A-type potassium current, respectively:

$$C_m \frac{dV_e}{dt} = -I_{\mathrm{Na}} - I_{\mathrm{NaP}} - I_{\mathrm{Kdr}} - I_{\mathrm{KA}} - I_{\mathrm{L}} - I_{\mathrm{GABA}} - I_{\mathrm{AMPA}} + I_{\mathrm{Inj}} + C_m\xi \qquad (5)$$

The currents in (5) are described by:

$$\begin{aligned}
I_{\mathrm{L}} &= g_{\mathrm{L}}(V_e - E_{\mathrm{L}})\\
I_{\mathrm{Na}} &= g_{\mathrm{Na}} m_\infty^3(V_e) h (V_e - E_{\mathrm{Na}})\\
I_{\mathrm{NaP}} &= g_{\mathrm{NaP}} p_\infty(V_e)(V_e - E_{\mathrm{Na}})\\
I_{\mathrm{Kdr}} &= g_{\mathrm{Kdr}} n^4 (V_e - E_{\mathrm{K}})\\
I_{\mathrm{KA}} &= g_{\mathrm{KA}} a_\infty^3 b (V_e - E_{\mathrm{K}})\\
I_{\mathrm{GABA}} &= g_{ie} s_{ie}^{\mathrm{tot}}(t)(V_e - E_{\mathrm{GABA}})\\
I_{\mathrm{AMPA}} &= g_{ee} s_{ee}^{\mathrm{tot}}(t)(V_e - E_{\mathrm{AMPA}})
\end{aligned} \qquad (6)$$

Here, the gating variables m, p and a are fast and are replaced by their asymptotic values m_∞, p_∞, and a_∞, respectively. The dynamics of the other gating variables are given by:

$$\begin{aligned}
\frac{dh}{dt} &= \frac{h_\infty(V_e)-h}{\tau_h}\\
\frac{dn}{dt} &= \frac{n_\infty(V_e)-n}{\tau_n}\\
\frac{db}{dt} &= \frac{b_\infty(V_e)-b}{\tau_b}
\end{aligned} \qquad (7)$$

The rate constants in the above equations are:

$$
\begin{aligned}
m_\infty &= \left[\exp\left(-\frac{V_e+30}{9.5}\right)+1\right]^{-1}\\
h_\infty &= \left[\exp\left(\frac{V_e+53}{7}\right)+1\right]^{-1}\\
\tau_h &= 0.37+2.78\left[\exp\left(\frac{V_e+40.5}{6}\right)+1\right]^{-1}\\
p_\infty &= \left[\exp\left(-\frac{V_e+40}{5}\right)+1\right]^{-1}\\
n_\infty &= \left[\exp\left(-\frac{V_e+30}{10}\right)+1\right]^{-1}\\
\tau_n &= 0.37+1.85\left[\exp\left(\frac{V_e+27}{15}\right)+1\right]^{-1}\\
a_\infty &= \left[\exp\left(-\frac{V_e+50}{20}\right)+1\right]^{-1}\\
b_\infty &= \left[\exp\left(\frac{V_e+80}{6}\right)+1\right]^{-1}\\
t_b &= 15 \text{ ms}
\end{aligned}
\tag{8}
$$

1.1.3 Noise

At each time step and for each neuron *independently*, the noise term $C_m\xi$ was drawn from a uniform distribution between $-\sqrt{6\lambda/_{dt}}$ and $\sqrt{6\lambda/_{dt}}$ (see table SI1).

1.2 Synapses

The equations for the connection type specific synaptic input current (AMPA or GABA) were given in (2) and (6) and have the general form:

$$I_{\text{syn}} = g_{kl}s_{kl}^{\text{tot}}(V - E_{\text{syn}}) \tag{9}$$

Here, g_{kl} is the unitary synaptic conductance (in mS/cm²) for connections from the presynaptic neurons of type k to a postsynaptic neuron of type l. Variable s_{kl}^{tot} represents the total input to the postsynaptic cell and depends on the network structure and the synaptic gating variables s of the presynaptic cells:

$$
\begin{aligned}
\frac{ds}{dt} &= \alpha F(V_{pre})(1-s) - \beta s\\
F(V_{pre}) &= \frac{1}{1+\exp(-\frac{V_{pre}-\theta_S}{\sigma_S})}
\end{aligned}
\tag{10}
$$

Parameters used in the equations can be found in Table SI1.

Parameter (unit)	Pyramidal cells	Interneurons
E_L (mV)	-70	-65
E_{Na} (mV)	55	55
E_K (mV)	-90	-90
E_{GABA} (mV)	0	0
E_{AMPA} (mV)	-75	-75
g_L (mS/cm^2)	0.02	0.1
g_{Na} (mS/cm^2)	24	35
g_{NaP} (mS/cm^2)	0.07	-
g_{Kdr} (mS/cm^2)	3	-
g_{KA} (mS/cm^2)	1.4	-
g_K (mS/cm^2)	-	9
C_m (μF/cm^2)	1	1
ϕ	-	5
λ (mV^2/ms)	0.06	0.02
Delay within area (ms)	1	1
Delay between areas (ms)	5	5
	**AMPA**	**GABA**
θ_s (mV)	-20	0
α (ms^{-1})	0.8	10
β (ms^{-1})	0.5	0.2
σ_s (mV)	2	2

Table SI1. Parameters for pyramidal cells, interneurons and synapses.

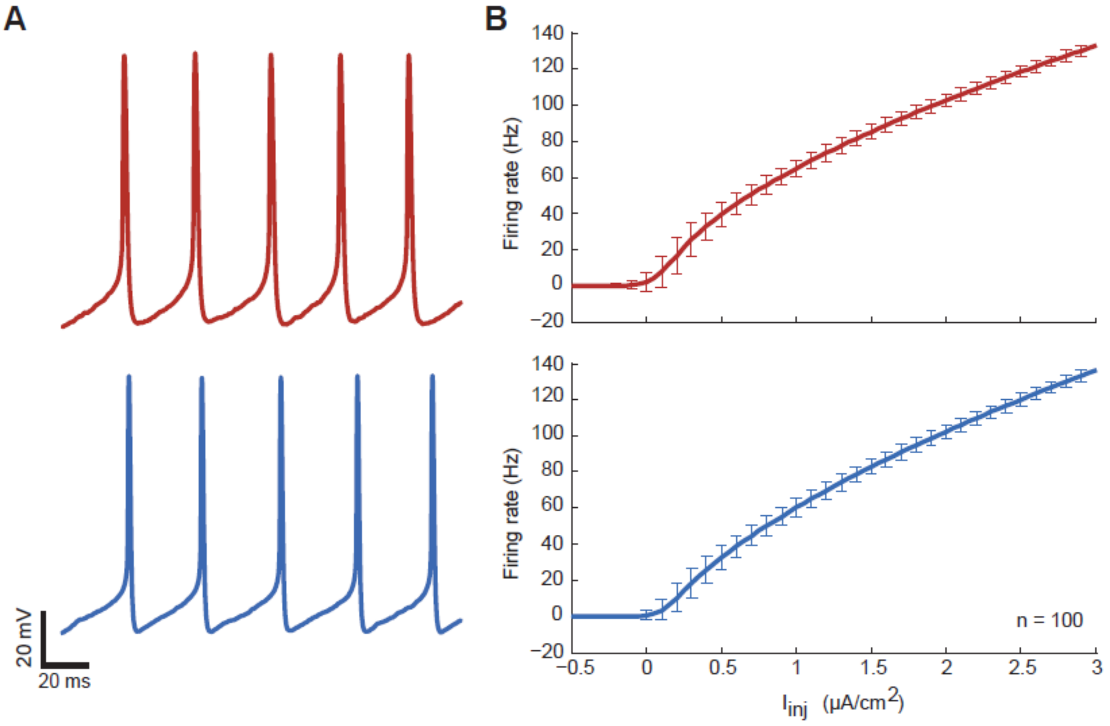

Figure SI1. Behavior of isolated neurons. A: Membrane potential traces of a pyramidal cell (red) and an interneuron (blue); B: fI-curve (firing rate vs. injected current) for both pyramidal cells (red) and interneurons (blue). We show the average (curves) and standard deviation (error bars) over 100 runs.

1.3 Pulsed inputs

The pulse current used in Figure 3 was modeled as:

$$I_{\text{pulse}} = g_{\text{pulse}} s_{\text{pulse}}(t - t_{\text{start}})(V - E_{\text{AMPA}}) \tag{11}$$

The variable s_{pulse} is an approximation of the s gating variable for AMPA-synapses given in Suppl. Equation 4. The dynamics of s were simplified by assuming fast dynamics of the voltage dependent opening (F variable), allowing for a steady state approximation of F and a subsequent simplification of the differential equation for s. We approximate the incoming spike by a 1 ms block pulse, so F=1 for 1 ms after the onset of the action potential and F=0 otherwise. The variable s_{pulse} is then described by:

$$s_{\text{pulse}} = \begin{cases} \frac{-\alpha}{\alpha+\beta} e^{-(\alpha+\beta)t} + \frac{\alpha}{\alpha+\beta} & \text{if } F = 1 \\ \frac{\alpha}{\alpha+\beta}\left(e^{\beta} - e^{\alpha}\right)e^{-\beta t} & \text{otherwise} \end{cases} \tag{12}$$

2 Supplementary Figures

2.1 Supplement to Result 3.1

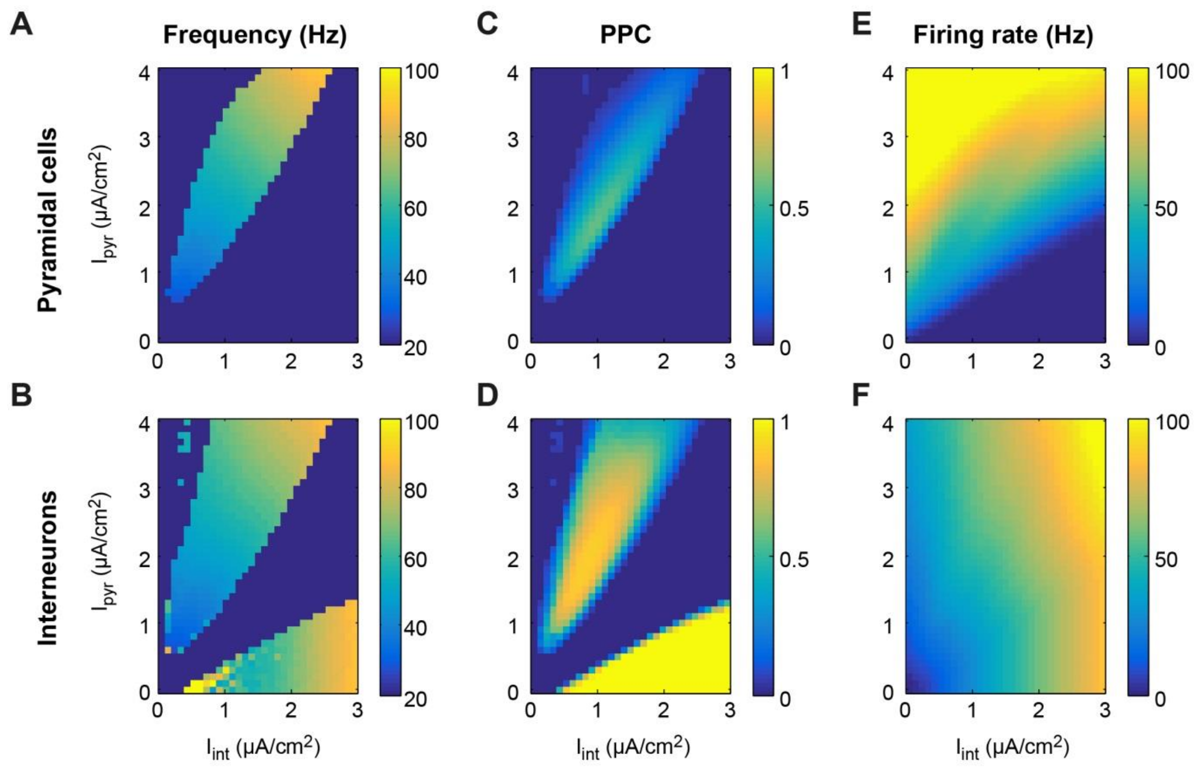

Figure SI2. Oscillation frequency (A and B), synchronization, as characterized by the PPC (C and D), and firing rate (E and F) of the single circuit reported in section 3.1 of the main text. The top row shows the measures based on the pyramidal cell spikes/spike density trace, while the bottom row is based on the interneuron population. Interneurons are more phase locked than pyramidal cells in the PING mechanism (left triangle), but the oscillation frequencies found based on the two cell types are identical. Interneurons show very strong phase locking in the ING region, as indicated by the high PPC values.

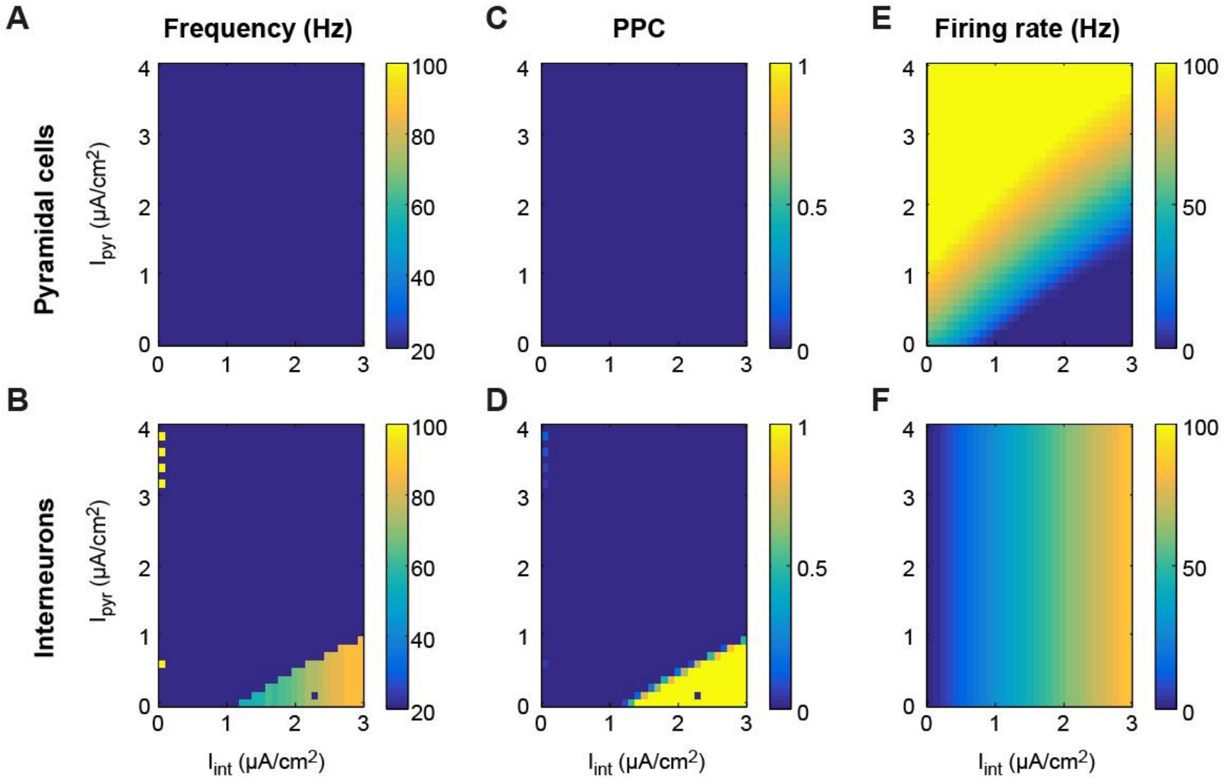

Figure SI3. As in Figure SI2: Oscillation frequency (A and B), PPC (C and D), and firing rate (E and F) of the single circuit in Figure 1 and Figure SI2, but without the connections from the pyramidal cells to the interneurons (interneuron to pyramidal cell connections remained intact). Top row: based on pyramidal cell spikes/spike density trace; bottom row: based on interneuron spikes/spike density traces. Without connections from the pyramidal cells to the interneurons, there is no PING synchronization, even though both cell types are active (except for the lower left corner where the pyramidal cells are suppressed). In the ING region, synchronization is unaltered. Data from one simulation.

2.2 Supplement to Result 3.2

Figure SI4 (next page). Synchronization of oscillatory activity in a feedforward network of two local circuits. A: Frequency difference between the two circuits (f_2 minus f_1), hence a positive difference indicates that circuit 2 oscillates at a higher frequency than circuit 1, while a negative difference indicates that circuit 1 oscillates at a higher frequency. A difference of 0 corresponds to synchrony, which agrees with the coherence reported in Fig 2 of the main text. B: Pairwise Phase Consistency between the pyramidal cells' spikes in circuit 2 and the pyramidal cell spike density (i.e. LFP) in circuit 1. The high PPC around the axis of the area of synchrony indicates strong synchronization between circuit 1 and 2; C: The PPC of the spikes of circuit 2 and the spike density in circuit 2 shows the same high values in the center of the Arnold tongue as the $PPC_{2\rightarrow1}$ in B, due to the high coherence between the LFPs of the two circuits. In the region of low coherence, circuit 2 shows internal synchronization, indicated by the extended region of intermediate synchronization. D: Standard deviation across the 5 runs of the coherence reported in Fig 2B, indicating variability is

highest near the edge of the Arnold tongue, where change in coherence is large; E & F: Standard deviations of the PPC between spikes in circuit 2 and LFP of circuit 1 (E) and between spikes and LFP from circuit 2 (F). The black lines in B, C, E and F indicate the PPC = 0.25 (thin) and PPC = 0.50 (thick) contours. G-H: Same analyses as before, but performed for the spike density of the interneuron population. Results for coherence (G) and phase difference (H) are qualitatively and similar to that of the pyramidal cell population. The only difference between the cell types occurs at high phase differences, where the phase difference for interneurons is lower than for pyramidal cells. This can be explained by the results in I: the interneurons in circuit 2 respond faster to inputs at high frequencies, as the drive coincides more and more with the inhibitory volley of the circuit. This biases the interneuron phase difference to lower values for high frequencies.

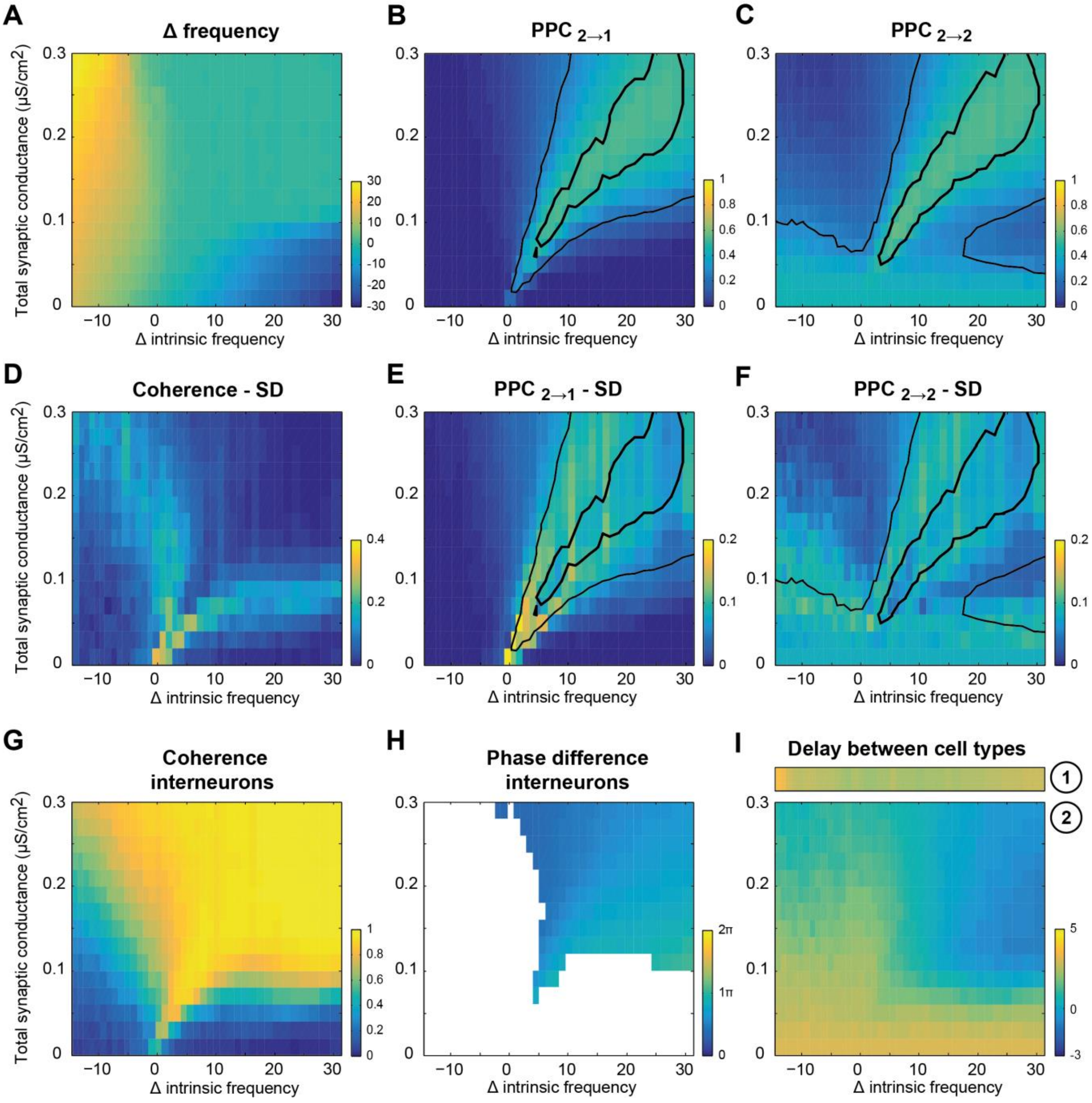

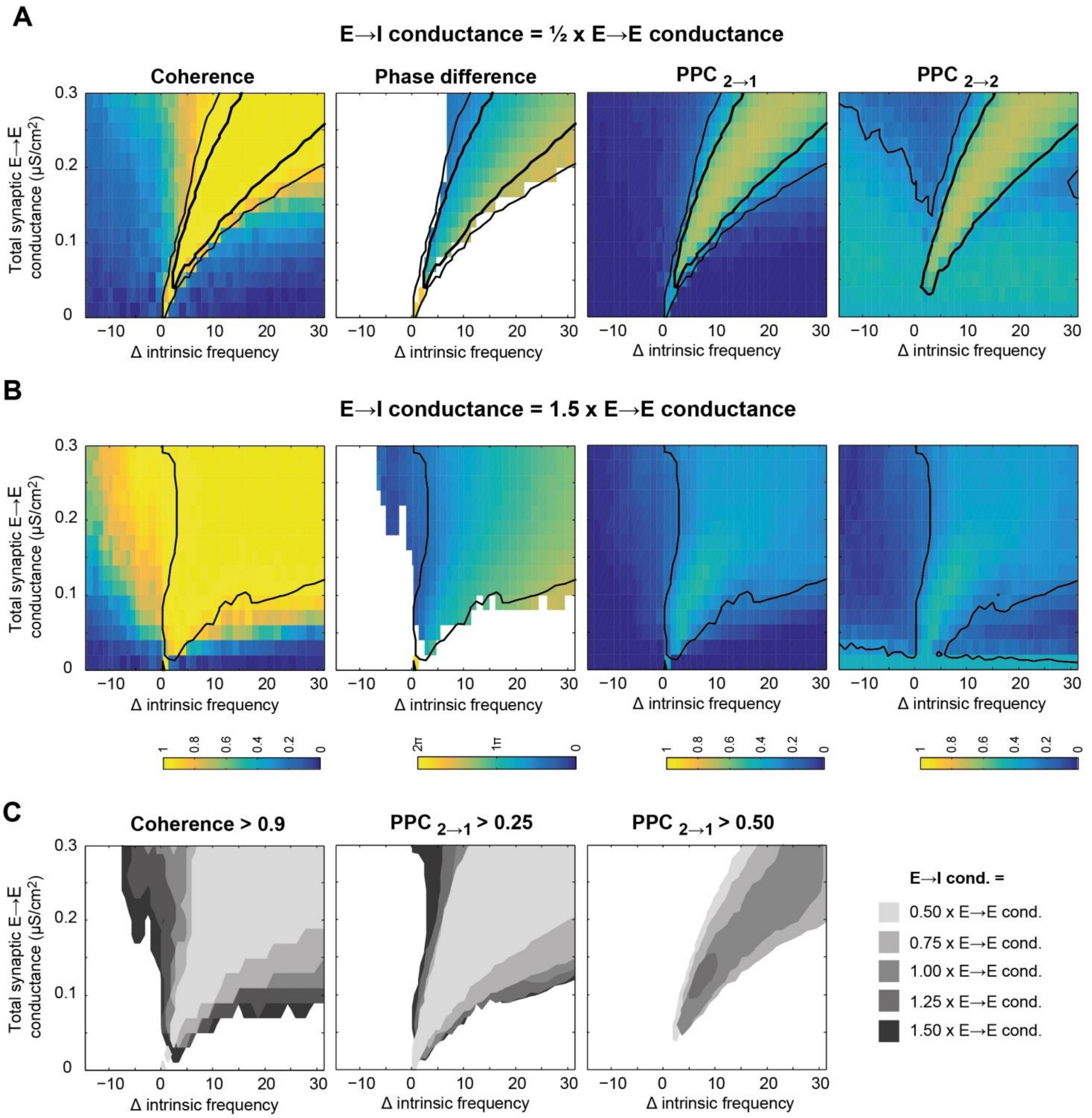

Figure SI5. The ratio between the amount of excitation (E) and inhibition (I) recruited by the projections from circuit 1 to circuit 2 determines the width of the Arnold tongue and the level of synchronization between the circuits. The total $E_1 \rightarrow E_2$ synaptic conductance was varied identical to the simulations in Figure 2 of the main text (y-axis), but the $E_1 \rightarrow I_2$ synaptic conductance was multiplied by a factor 0.5 in panel A, and 1.5 in panel B. Panel C summarizes the area of high (>0.9) coherence for 5 ratios, as well as the PPC between circuit 1 LFP and circuit 2 spikes. More inhibition led to wider Arnold tongues (C, middle: contours widen), but lower PPCs between the circuits (C, right: contours narrow). Data shown are from one simulation.

2.3 Supplement to Result 3.3

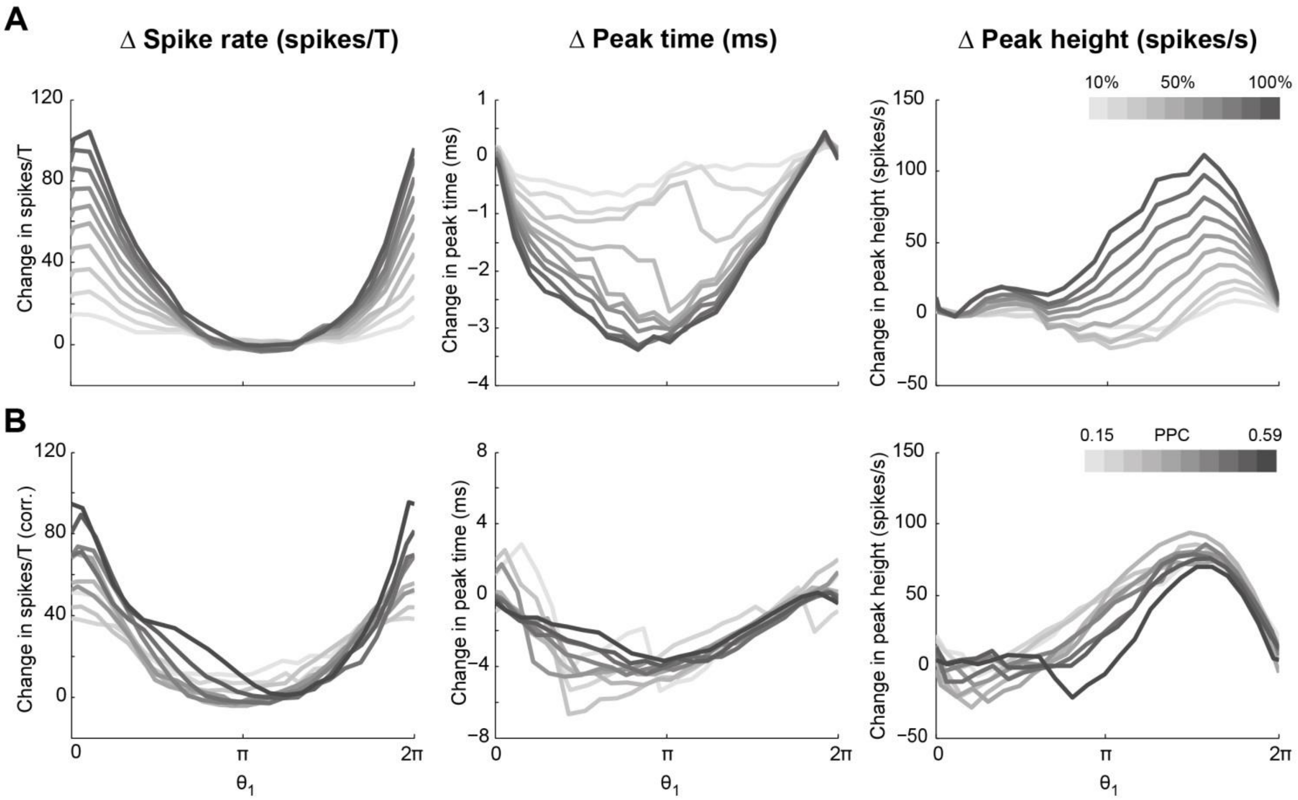

Figure SI6. The effect size of pulse application to a circuit for different numbers of excitatory neurons targeted (A) and different levels of within-circuit synchronization (B). **A:** The size of the assessed changes in spikes rate, peak time and peak height varied in a predictable way with the number of neurons that receive the pulse. The phase dependency did not change with number of neurons targeted. B: The size of change in spike rate depended on the PPC of the receiving circuit, with low PPC giving rise to smaller changes (the data are corrected for changes in intrinsic spike rate between different levels of synchronization). Peak time changed more for lower PPC, while peak height changes did not depend on PPC. Figures represent the average across 5 simulations.

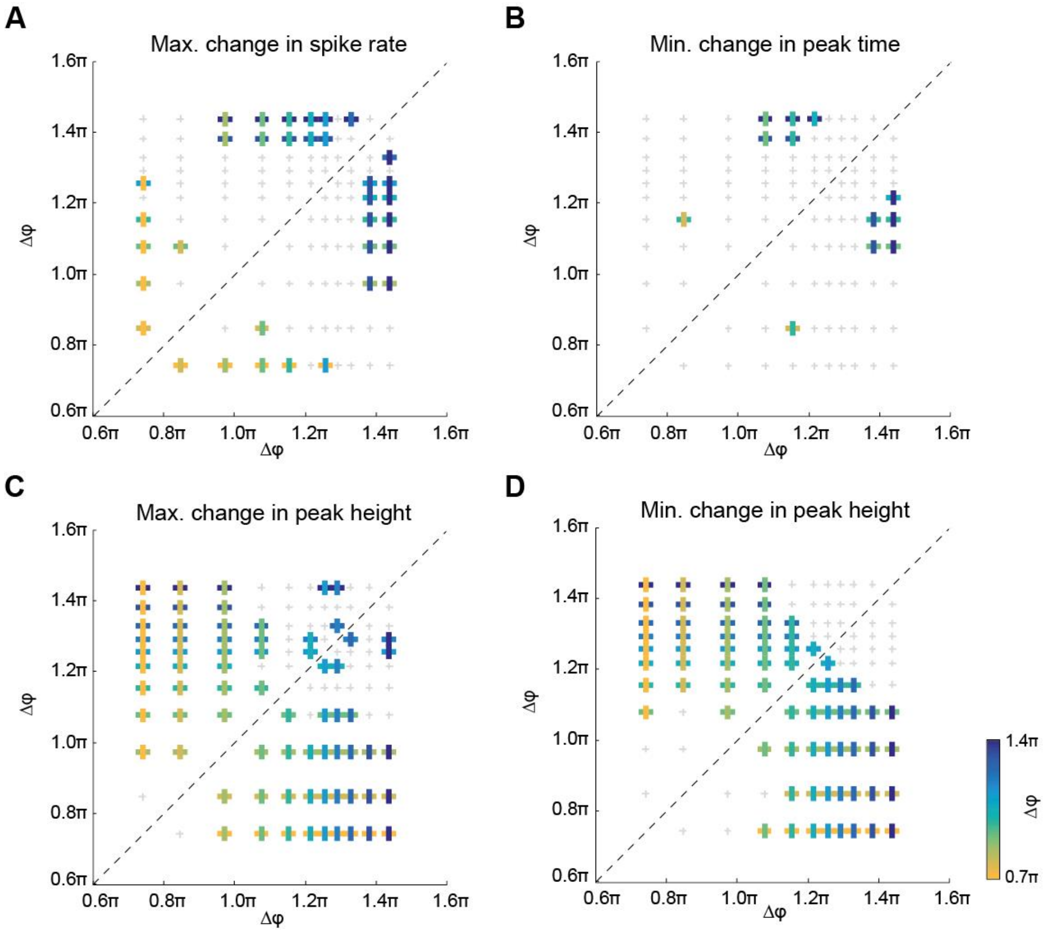

Figure SI7. Comparison of maximal or minimal effect sizes in the receiving circuit of a feedforward network after a pulse is delivered to the sending circuit (i.e. peaks/troughs of the lines in Figure 3D), for different phase differences between the circuits. Each cross compares the effect sizes of two phase differences, indicated by the x- and y-axes. Fat crosses indicate significant differences ($p<0.05$ at a paired t-test, False Discovery Rate corrected, $n=10$), while small gray crosses indicate absence of significance. Color code as in Figure 3 of the main text, to aid comparison. Note that the paired t-test is undirected and hence the figures are symmetric in the diagonal. Effect sizes were significantly different between most phase differences for peak height. For spike rate, the higher and lower phase differences differed from the middle phase differences, but not each other. Peak time showed few significant differences, in agreement with the correlations in Table SI3.

Table SI3. Circulair-linear correlations between phase difference between the circuits in the network and the maximal or minimal effect sizes of a pulse to the first circuit on the second circuit.

Measure	Correlation coefficient (ρ)	p-value
Max. spike rate	0.49	<0.0001
Min. peak time	0.16	0.23
Max. peak height	0.57	<0.0001
Min. peak height	0.73	<0.0001

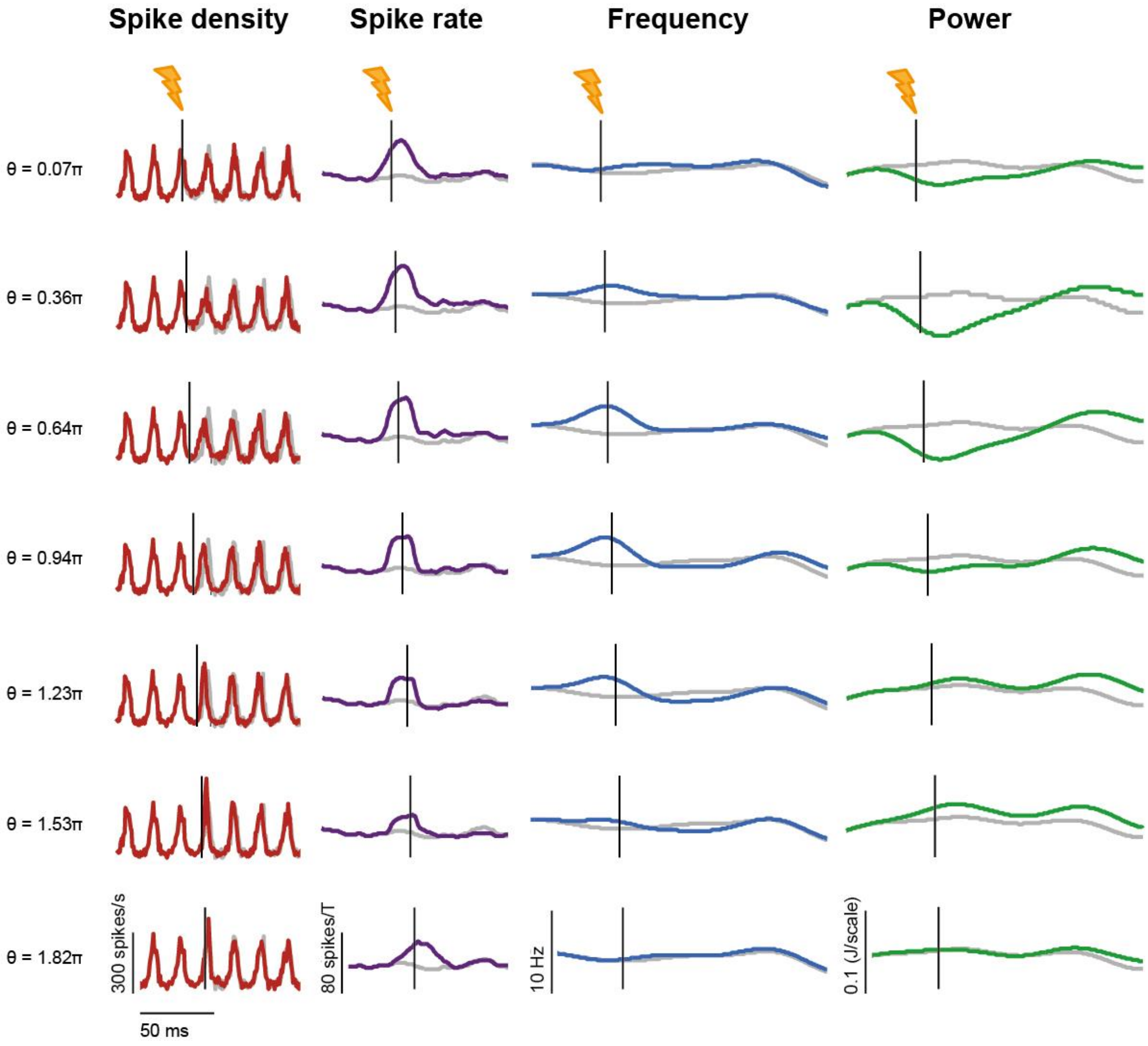

Figure SI8. Examples traces of time-resolved spike rate, frequency and power with (colored lines) and without (gray lines) pulsed inputs (yellow lines) at different phases in the ongoing oscillation. See Methods section of the main text for a description of how the signals were obtained. Compare with Fig 3 of the main text.

2.4 Supplement to Result 3.4

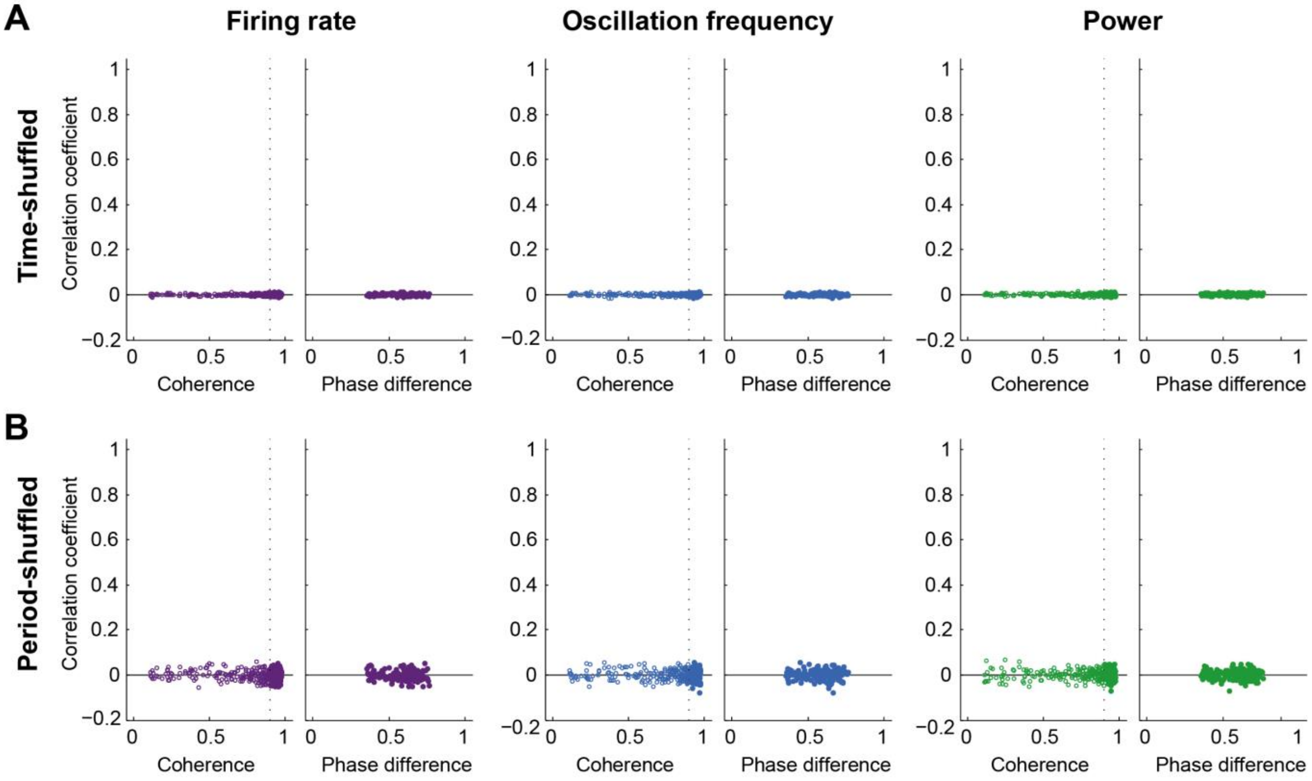

Figure SI9. Correlation coefficient between firing rate (purple), frequency (blue) and power (green) traces of circuit 1 and circuit 2 (same data as Figure 4 of the main text), after two shuffling procedures. A: Time-shuffling: Traces from circuit 2 were randomly shuffled in time. This procedure reduced the correlation coefficient to around 0 (no correlation). B: Period-shuffling: Traces of circuit 2 were cut into windows of one oscillation period and the windows were randomly shuffled, while maintaining the temporal structure within oscillation periods. Despite the presence of short-term temporal structure, the resulting correlation coefficients between the circuits was reduced to around 0, indicating that the correlation coefficient is not dominated by oscillatory structure in the traces.

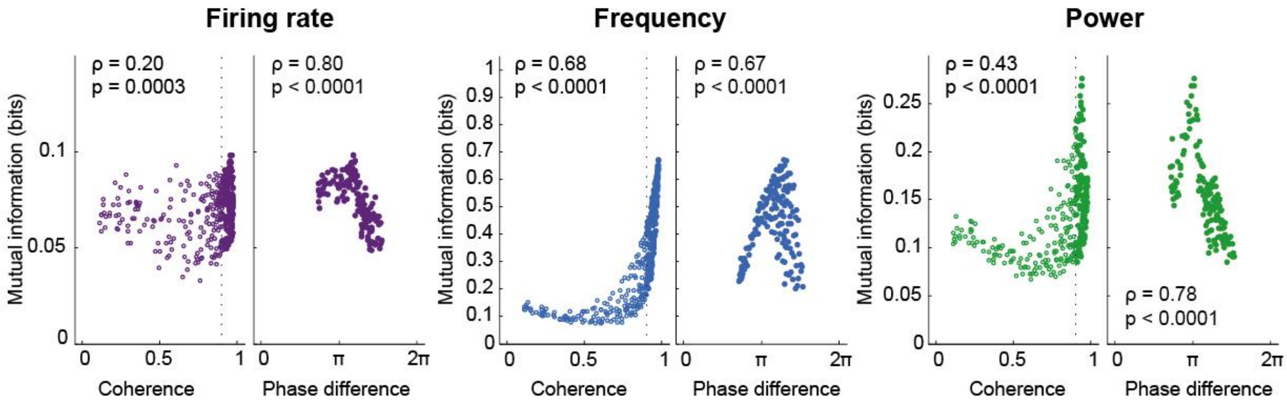

Figure SI10. Mutual information between circuit 1 and circuit 2 changed with coherence and phase. Same data as in Fig 4 of the main text, but now analyzed using Mutual Information with 6 bins.

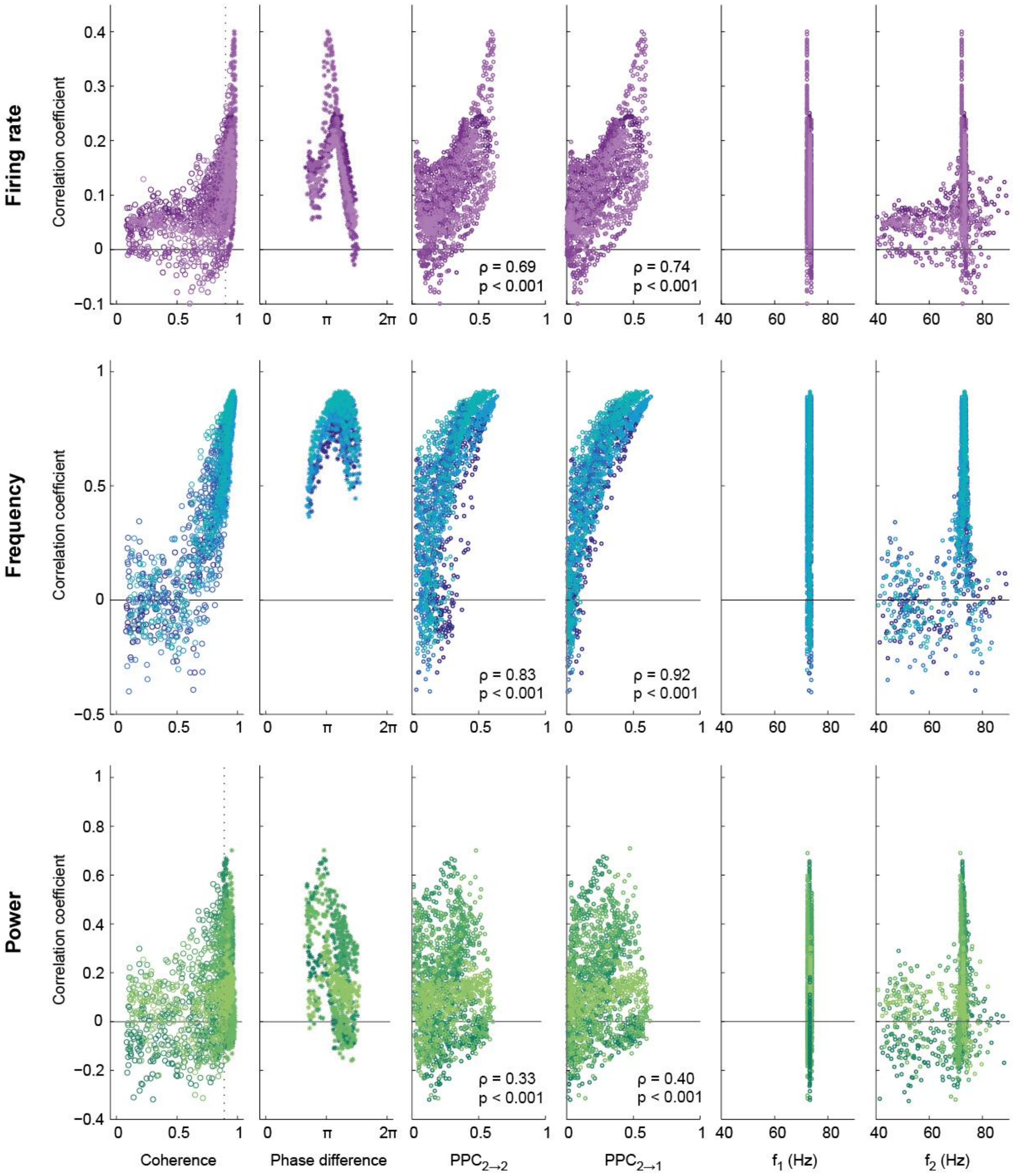

Figure SI11. Correlation coefficient between firing rate (purple), frequency (blue) and power (green) traces of circuit 1 and circuit 2 for 5 simulation runs (saturation), against a coherence and phase difference (same data as Figure 4 of the main text), as well as against the inter-circuit and intra-circuit PPCs and the oscillation frequencies of circuit 1 and 2. Variability between runs was high for power due to differences in offset, though individual runs all show coherence and phase dependent information transfer. PPC and frequency were related to coherence and phase, and are therefore also correlated to the efficiency of information transfer between the circuits.

2.5 Supplement to Result 3.5

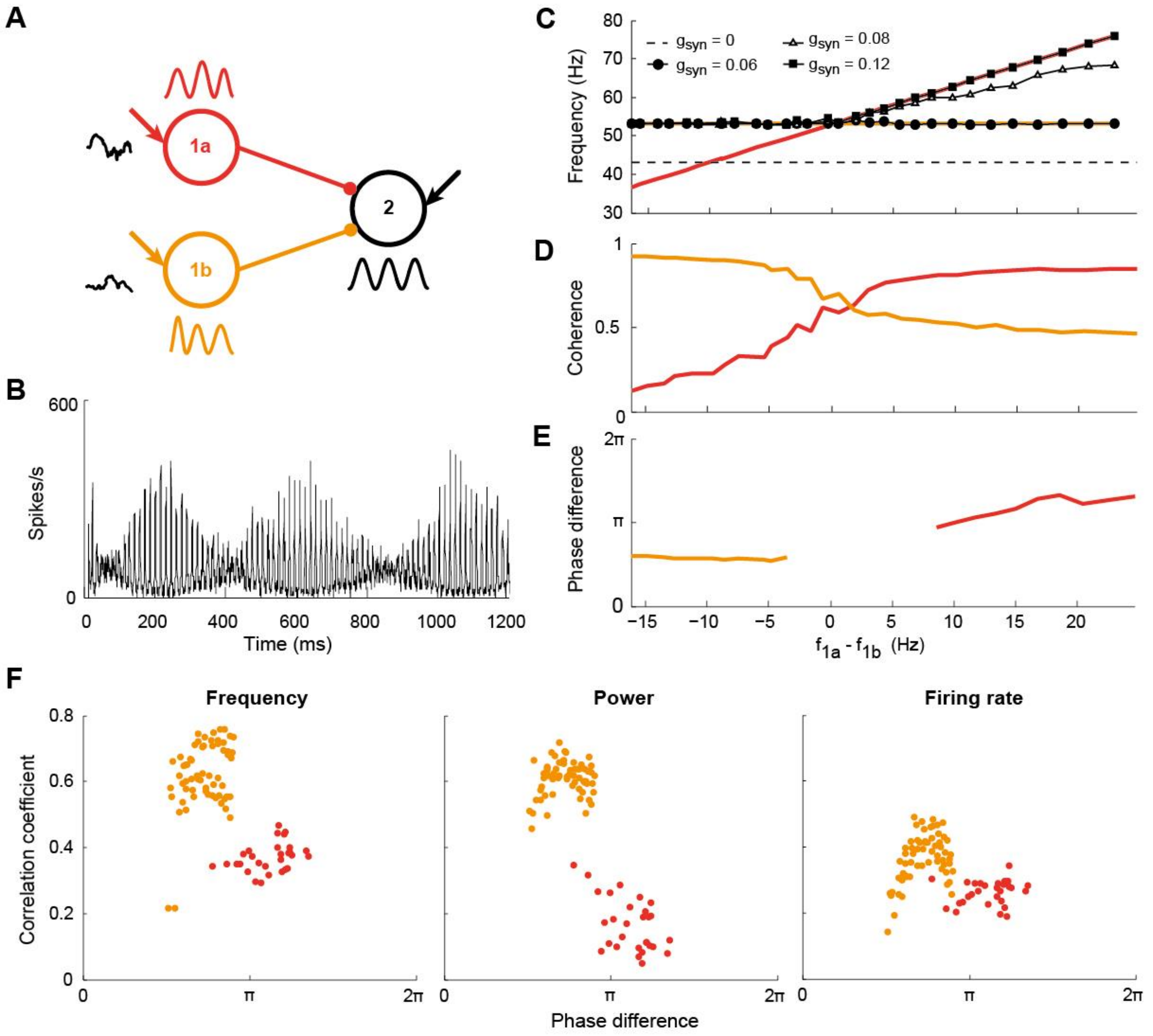

Figure SI12. Coherence and phase allow for switching between, and selection of, inputs in a network with multiple senders. A: Model network with two senders with feedforward projections to a single receiver. All circuits were placed in a dynamical regime with intrinsic oscillations (green, purple and black traces) by choosing the appropriate level of depolarizing currents, in addition the senders received noise currents that were independent and uncorrelated for the two senders. Color code throughout the figure: purple indicates circuit 1a or the relation between 1a and 2; green indicates circuit 1b or the relation 1b-2; black indicates circuit 2. B: When both of the senders had approximately the same intrinsic frequency, the receiver showed interference, producing a beat-like pattern with periods of high coherence and phase precession interleaved with periods of asynchrony. C: When the two senders had different intrinsic frequencies, the sender with the highest frequency dominated the receiver, as long as the synaptic strength was sufficient to drive the receiver. The black curves indicate the frequency of the receiving circuit for different values of projection strength from zero (dashed line) to g_{syn}=0.06, 0.08, and 0.12 $\mu S/cm^2$ indicated by circles, triangles and squares, respectively. D-E: In a state of interference, instantaneous coherence (D) and phase difference (E) between the senders and receiver determined the information transferred (F),

quantified by the correlation coefficient of all data binned into coherence/phase bins. Information transfer only was high for ‘good’ phase relations between relevant sender and receiver, which was around 0.8π for this network, a value that was not reached by sender 1a. Data shown are from one simulation.

2.6 Supplement to Discussion

The analysis of synchrony between two circuits (Fig. 2 of the main text) was repeated for circuits oscillating through an ING mechanism (SI section 2.6.3). To this end, an ING-circuit was set up (SI section 2.6.1) and its behavior was characterized (SI section 2.6.2). The behavior of a feedforward network of two circuits oscillating through an ING mechanism is shown in section 2.6.3. The data shown are from one simulation.

2.6.1 Adjustments to the model

To allow for a wider range of ING conditions than available in the model used to study PING, we adjusted the connection probabilities of the circuit model: The connection probability between interneurons was increased, while the connection probabilities between pyramidal cells and interneurons and vice versa were reduced. This allowed for an ING synchronization while the pyramidal cells are still active, a regime that was too small to study in the model in the main text.

In addition, we allowed for direct inhibitory connections between two circuits in the network setup, while the PING model in de main text was only connected by excitatory connections. The connection parameters for the ING model are given in Table SI4.

Table SI4: Connection probabilities and unitary synaptic strengths for the connections in the ING network model.

Connection type	Connection probability (%)	Unitary synaptic strength (μS/cm^2)
E to E within circuit	10	1.2
E to I within circuit	20	1.0
I tot I within circuit	40	12
I to E within circuit	40	5.0
E to E between circuits	5	0 - 15
E to I between circuits	10	0 - 7.5
I to I between circuits	10	0 - 7.5
I to E between circuits	10	0 - 7.5

2.6.2 Characterization of the ING circuit

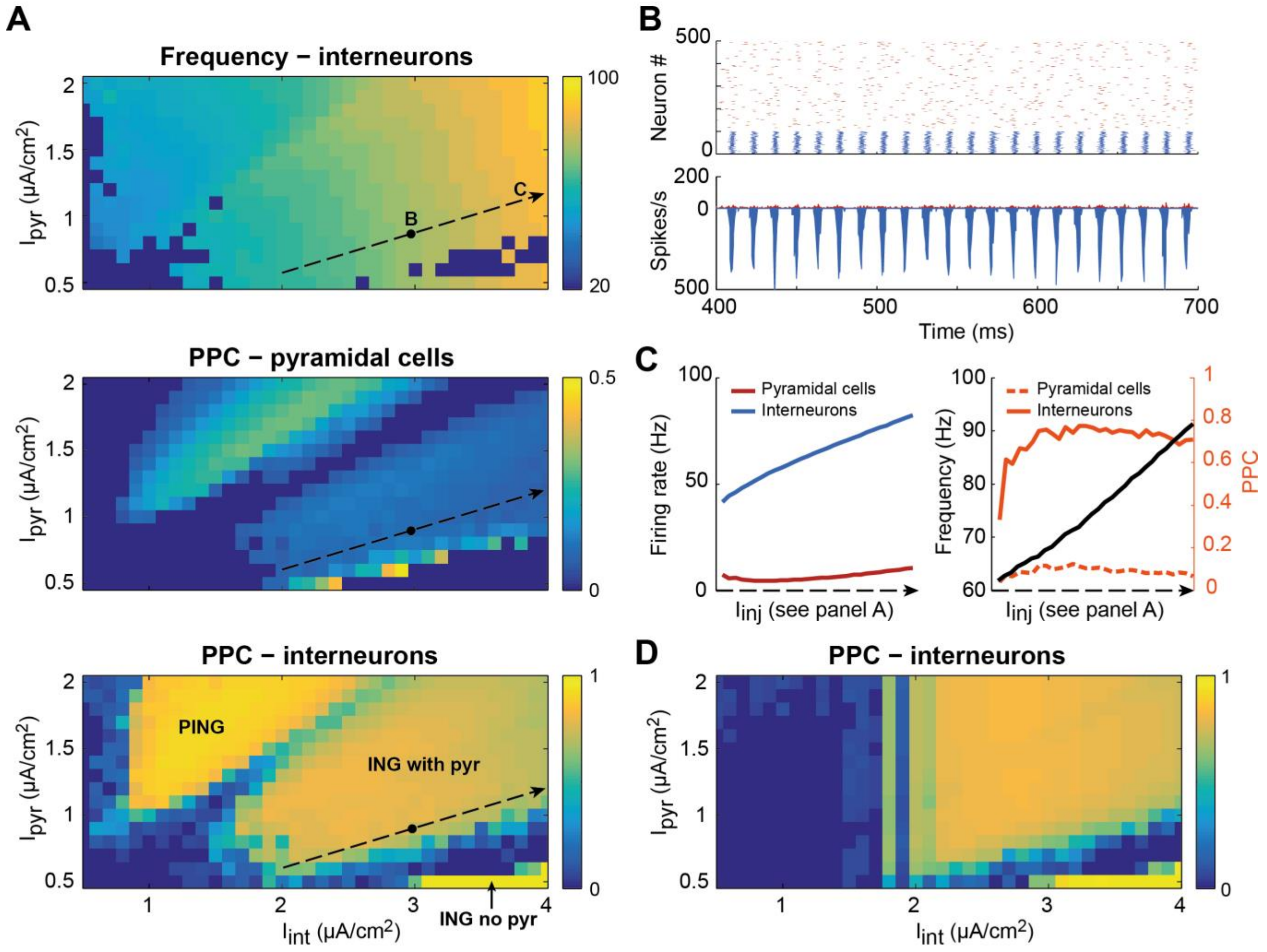

Figure SI13. Characterization of the ING model. A: As in Figure 1 in the main text, the depolarizing drive to the interneurons and pyramidal cells in the circuit was varied. Note that the ranges are different from those in Figure 1C. Oscillation frequency (top) and PPC of the pyramidal cells (middle) and interneurons (bottom) is shown. As for the PING model, there is a triangular region with high PPC for both cell types in the left top corner (indicated in the bottom panel), which is caused by synchronization through the PING mechanism (compare with D). In addition, there is a large region of high interneuron PPC, but with lower pyramidal cell PPC. This additional region is caused by ING synchronization, while still allowing for pyramidal cell activity. Furthermore, there is a region of ING activity without pyramidal cell activity, which was also shown in the model in the main text. B: Example rastergram (top) and spike density plot (bottom) of the input condition indicated by a dot in A (top panel). C: Change of firing rates (left) and frequency and PPC (right) along the line indicated in A. These conditions are used for the simulations for Figure SI14. D: Same drive conditions as in A, but with no connections from pyramidal cells to interneurons. This reduces synchronization in the PING region, but not the ING regions, indicating that the main source of synchronization in these regions are the recurrent connections between interneurons.

2.6.3 Feedforward network of two ING-circuits

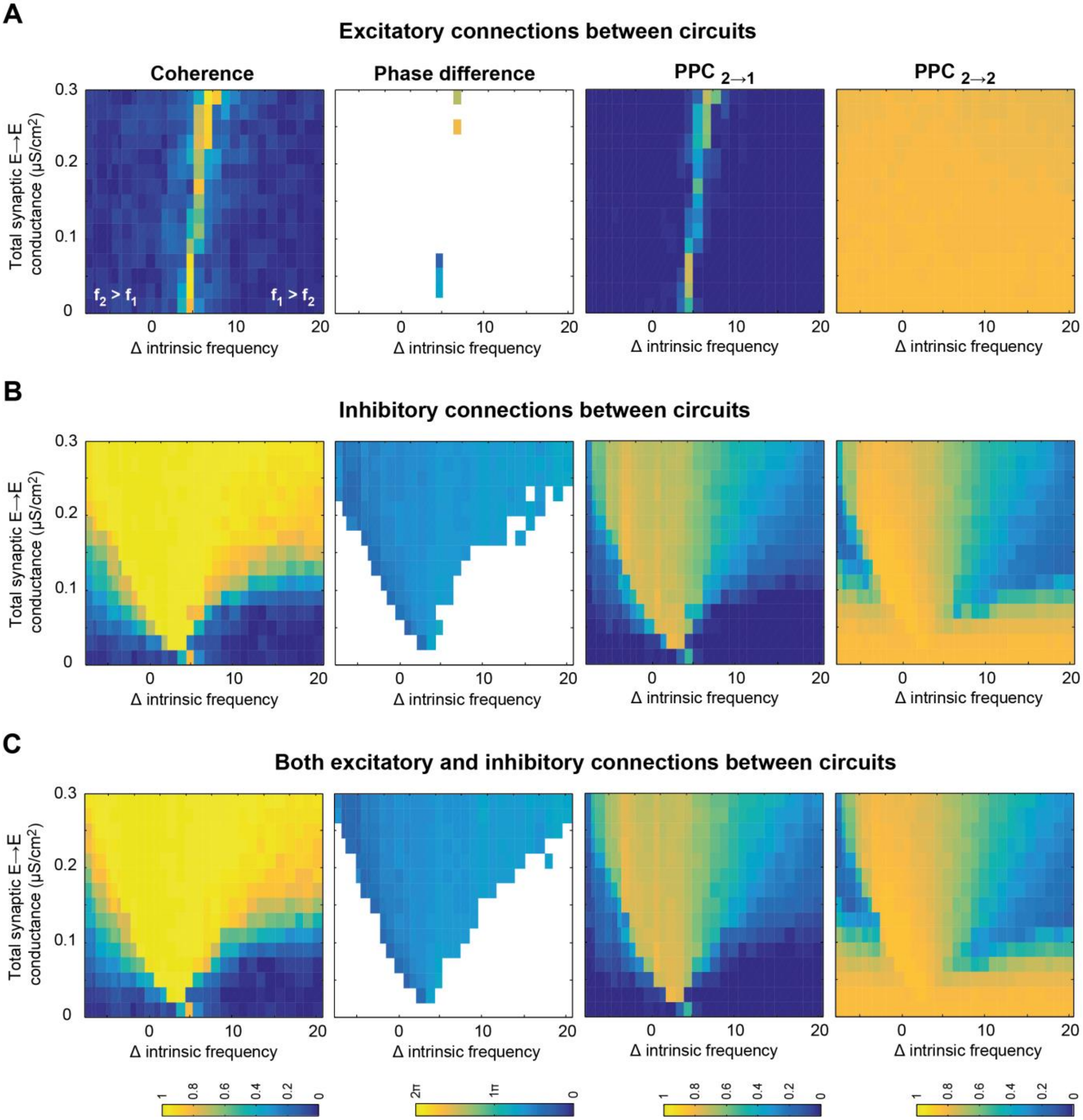

Figure SI14. Analysis of synchrony and phase between two circuits oscillating in ING regimes. Input conditions for circuit 1 are as shown in Figure SI13C, while for circuit 2 $I_{\text{int}} = 2.76\ \mu\text{A/cm}^2$ and $I_{\text{pyr}} = 1.57\ \mu\text{A/cm}^2$. Unlike the main text, the analyses presented here were performed on data from the interneuron population, as the pyramidal cell activity was low and therefore less reliable. However, pyramidal cell data showed qualitatively similar results. Coherence, phase difference and PPC of spikes from circuit 2 to LFP activity of circuit 1 ($\text{PPC}_{2\to1}$) and to LFP activity of circuit 2 ($\text{PPC}_{2\to2}$) for three different feedforward projections: only excitatory connections to pyramidal cells and interneurons in the receiving circuit (A); only inhibitory connections to both cell types in the receiving circuit (B); both excitatory and inhibitory connections to both cell types in the receiver (C).
